# Homeodomain-interacting protein kinase promotes tumorigenesis and metastatic cell behavior

**DOI:** 10.1242/dmm.031146

**Published:** 2018-01-01

**Authors:** Jessica A. Blaquiere, Kenneth Kin Lam Wong, Stephen D. Kinsey, Jin Wu, Esther M. Verheyen

**Affiliations:** Department of Molecular Biology and Biochemistry, Centre for Cell Biology, Development and Disease, Simon Fraser University, Burnaby, British Columbia, Canada V5A 1S6

**Keywords:** Hipk, Metastasis, Tumor, Cancer

## Abstract

Aberrations in signaling pathways that regulate tissue growth often lead to tumorigenesis. Homeodomain-interacting protein kinase (Hipk) family members are reported to have distinct and contradictory effects on cell proliferation and tissue growth. From these studies, it is clear that much remains to be learned about the roles of Hipk family protein kinases in proliferation and cell behavior. Previous work has shown that *Drosophila* Hipk is a potent growth regulator, thus we predicted that it could have a role in tumorigenesis. In our study of Hipk-induced phenotypes, we observed the formation of tumor-like structures in multiple cell types in larvae and adults. Furthermore, elevated Hipk in epithelial cells induces cell spreading, invasion and epithelial-to-mesenchymal transition (EMT) in the imaginal disc. Further evidence comes from cell culture studies, in which we expressed *Drosophila* Hipk in human breast cancer cells and showed that it enhances proliferation and migration. Past studies have shown that Hipk can promote the action of conserved pathways implicated in cancer and EMT, such as Wnt/Wingless, Hippo, Notch and JNK. We show that Hipk phenotypes are not likely to arise from activation of a single target, but rather through a cumulative effect on numerous target pathways. Most *Drosophila* tumor models involve mutations in multiple genes, such as the well-known Ras^V12^ model, in which EMT and invasiveness occur after the additional loss of the tumor suppressor gene *scribble.* Our study reveals that elevated levels of Hipk on their own can promote both hyperproliferation and invasive cell behavior, suggesting that Hipk family members could be potent oncogenes and drivers of EMT.

## Abstract (precis)

**Summary:** The protein kinase Hipk can promote proliferation and invasive behaviors, and can synergize with known cancer pathways, in a new *Drosophila* model for tumorigenesis.

## INTRODUCTION

A number of evolutionarily conserved signaling pathways are used reiteratively during development to control the growth of healthy organs and tissues. Genetic aberrations in pathway components can lead to dysregulated growth signals, often resulting in uncontrolled proliferation and tumorigenesis. With time and further genetic changes, tumor cells can progress into a metastatic state by undergoing epithelial-to-mesenchymal transition (EMT), enabling cells to leave the primary tumor site and travel to other locations in the body (reviewed by Thiery et al., 2009). Many of the cellular markers and processes involved in vertebrate tumorigenesis are conserved in *Drosophila*, which has been used for decades to study developmental signaling pathways and has been key in revealing molecular functions of human disease and cancer-related genes (Brumby and Richardson, 2005; Gonzalez, 2013; Potter et al., 2000; Rudrapatna et al., 2012). Tissue and organ growth are often studied using the larval imaginal discs, which are epithelial sacs composed primarily of a pseudo-stratified columnar monolayer (Aldaz and Escudero, 2010). Discs undergo extensive proliferation, with subsequent patterning and differentiation to form adult structures, which requires the same key signaling pathways needed for human development and growth (Brumby and Richardson, 2005; Gonzalez, 2013; Herranz et al., 2016; Miles et al., 2011; Rudrapatna et al., 2012; Sonoshita and Cagan, 2017). Low genetic redundancy paired with powerful genetic manipulation tools make *Drosophila* an excellent system for the study of tumorigenesis and metastasis.

Numerous signaling pathways have been implicated in the development of tissue overgrowth and/or metastatic behavior in the fly. The majority of these studies have described tumor models that require the combination of multiple genetic aberrations in order to manifest hyperproliferation coupled with invasive behaviors. The earliest metastasis model involved activated Ras combined with loss of the tumor suppressor *scribble* (Pagliarini and Xu, 2003). Notch pathway activation coupled with alterations in histone epigenetic marks also led to a *Drosophila* tumor model (Ferres-Marco et al., 2006). Subsequent studies have identified further factors involved in both Ras- and Notch-driven tumorigenesis (Doggett et al., 2015). Other tumor studies involve Epidermal growth factor receptor (Egfr) signaling (Herranz et al., 2012) and the Sin3A histone deacetylase (HDAC) (Das et al., 2013). The Hippo pathway is a potent tumor suppressor pathway that is required to prevent hematopoietic disorders (Milton et al., 2014). Activated JAK/STAT signaling causes leukemia-like hematopoiesis defects in *Drosophila* (Harrison et al., 1995; Luo et al., 1997).

Homeodomain-interacting protein kinases (Hipk) are evolutionarily conserved, and vertebrates possess Hipk1-Hipk4, whereas *Drosophila* and *Caenorhabditis* *elegans* have only one Hipk each. Hipk family members are expressed in dynamic temporal and spatial patterns, highlighting their important roles during development (reviewed by Blaquiere and Verheyen, 2017). Hipk protein levels are highly regulated by post-translational modification and proteasomal degradation (Saul and Schmitz, 2013). Hipk family members are reported to have distinct and contradictory effects on cell proliferation and tissue growth. Overexpressing *Drosophila* Hipk causes tissue overgrowths in the wing, eye and legs in a dose-dependent manner (Chen and Verheyen, 2012; Lee et al., 2009a; Poon et al., 2012). In *C. elegans*, Hpk-1 promotes proliferation of the germline cells, and loss of *hpk-1* reduces the number of proliferating cells and size of the mitotic region (Berber et al., 2013). *Hipk2*^−/−^ mice have growth deficiencies, and 40% die prematurely (Chalazonitis et al., 2011; Sjölund et al., 2014; Trapasso et al., 2009). In normal human skin, Hipk2 protein expression is enriched in basal proliferating cells, whereas it is undetectable in nonproliferating cells (Iacovelli et al., 2009), and expression is reactivated when cells are stimulated to proliferate, suggesting a close link between Hipk protein function and cell proliferation. Mouse embryo fibroblasts (MEFs) from *Hipk2*^−/−^ knockout mice show reduced proliferation (Trapasso et al., 2009), whereas another study claimed that such cells proliferated more than wild type (Wei et al., 2007). From these studies, it is clear that much remains to be learned about the roles of Hipk family protein kinases in proliferation and cell behavior.

Hipks regulate numerous signaling pathways required for the development of healthy tissues (Fig. S1; reviewed by Blaquiere and Verheyen, 2017). Both *Drosophila* and vertebrate Hipks can modulate Wnt signaling in many ways (Hikasa and Sokol, 2011; Hikasa et al., 2010; Kuwahara et al., 2014; Lee et al., 2009b; Louie et al., 2009; Shimizu et al., 2014; Swarup and Verheyen, 2011; Wu et al., 2012). Hipk proteins modulate the Hippo pathway in *Drosophila*, which is an essential conserved signaling pathway regulating tissue and organ growth (Chen and Verheyen, 2012; Poon et al., 2012). Yki activity requires Hipk, as *hipk* loss of function can suppress the effects of constitutively active Yki (Yki^S168A^). Hipks have also been shown to regulate Jun N-terminal kinase (JNK) signaling in numerous contexts (Hofmann et al., 2003, 2005; Huang et al., 2011; Lan et al., 2007, 2012; Rochat-Steiner et al., 2000; Song and Lee, 2003; Chen and Verheyen, 2012). Hipk is required for the full effect of JAK/STAT signaling, because loss of *hipk* through somatic clonal analysis causes loss of Stat92E-GFP reporter and, furthermore, loss of *hipk* can suppress lethality and tumor frequency in the constitutively active *hop^Tum−L^* allele (Blaquiere et al., 2016 preprint).

Hipk2 is the best-characterized vertebrate Hipk family member. Studies in cell culture and cancer samples reveal conflicting results (Blaquiere and Verheyen, 2017). For example, Hipk2 acts as a tumor suppressor in the context of p53-mediated cell death after lethal DNA damage (Hofmann et al., 2013), and reduced expression of Hipk proteins is seen in several cancer types (Lavra et al., 2011; Pierantoni et al., 2002; Ricci et al., 2013; Tan et al., 2014). By contrast, Hipk2 is elevated in certain cancers, including cervical cancers, pilocytic astrocytomas and colorectal cancer cells, and in other diseases, such as thyroid follicular hyperplasia (Al-Beiti and Lu, 2008; Cheng et al., 2012; D'Orazi et al., 2006; Deshmukh et al., 2008; Jacob et al., 2009; Lavra et al., 2011; Saul and Schmitz, 2013; Yu et al., 2009). Human Hipk1 is also found at elevated levels in certain cancer cell lines and tissue samples (Kondo et al., 2003; Rey et al., 2013).

Although it is known that *Drosophila* Hipk is a strong inducer of tissue growth, its role in tumorigenesis is less well understood. We were intrigued to test whether Hipk contributes to this process in *Drosophila*, because of the genetic simplicity of this model system. Using various techniques, we provide evidence that elevation of *hipk* can lead to neoplasia characterized by cell invasiveness. Hipk expression drives numerous cellular changes that are hallmarks of EMT. Hipk-expressing cells can migrate through tissues, disrupt the basement membrane to exit tissues and express mesenchymal markers. Our findings are significant in that Hipk alone can promote proliferation and invasive behavior that has been previously described to arise from the perturbation of multiple pathways. We propose that *Drosophila* Hipk has potent oncogenic properties, and that Hipk can exert such an effect through promotion of its multiple target pathways.

## RESULTS

### Elevated Hipk leads to overgrowths and masses

**Fig. 1.**
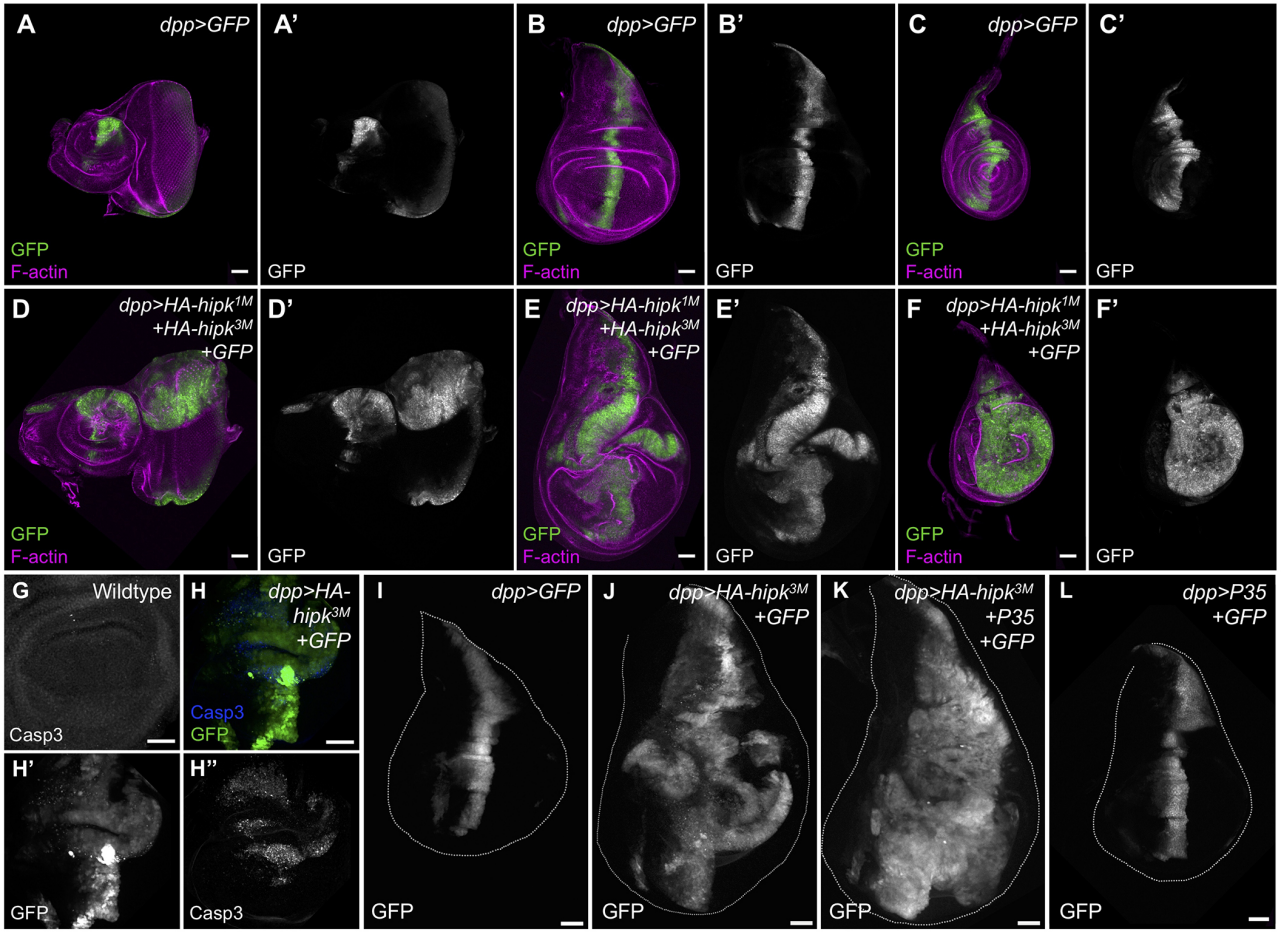
**Hipk induces overgrowths in *Drosophila* imaginal discs.** Control eye-antennal (A), wing (B) and leg (C) imaginal discs stained for actin to reveal tissue morphology and GFP to reveal the *dpp-Gal4* expression domain. Expression of two copies of wild-type *HA-hipk* (*HA-hipk^3M^* and *HA-hipk^1M^*) within the *dpp* domain leads to overgrown eye-antennal (D), wing (E) and leg (F) imaginal discs. (G) A control wing disc pouch stained for Casp3. (H) Casp3 is autonomously induced in *dpp>HA-hipk^3M^+GFP* wing discs. (I) A control wing imaginal disc showing the *dpp-GAL4* expression domain marked by GFP. (J) *dpp>HA-hipk^3M^+GFP* leads to overgrown wing discs. (K) Loss of cell death in *dpp>HA-hipk^3M^+GFP+P35* discs worsens *hipk*-induced overgrowths, whereas (L) *dpp>GFP+P35* appears normal. Scale bars: 50 μm.

To study the implications of elevated *hipk*, we used the GAL4-UAS system to overexpress Hipk in a variety of cell types. Using a combination of growth at different temperatures (which affects the potency of the GAL4 transcription factor) and copy number of UAS-transgenes, we have generated a range of Hipk overexpression phenotypes. Use of the *dpp-Gal4* driver to express two copies of Hipk at 25°C caused dramatic overgrowth of eye, wing and leg imaginal discs, characterized by tissue folds and protrusions (Fig. 1A-F′). We made use of GFP labeling to mark the *hipk-*overexpressing cells, allowing us to visualize their behavior (Fig. 1D-F′) in comparison with wild-type cells (*dpp>GFP*; Fig. 1A-C′). Coexpression of *hipk* and *GFP* at 29°C (*dpp>HA-hipk^3M^+GFP*) led to overgrown wing discs (Fig. 1J). Staining for cleaved Caspase 3 (Casp3) revealed that cell death was autonomously induced within the *hipk-*expressing discs (Fig. 1H-H″; Fig. S2A). When we used *P35* expression to block caspase-dependent cell death, cells within the Dpp domain expanded substantially and occupied almost the entire *dpp>HA-hipk^3M^+P35+GFP* discs (Fig. 1K), whereas *dpp>P35* alone had no effect in this context (Fig. 1L). This implies that Hipk can induce both cell death and abundant proliferation to induce the gain-of-function phenotypes.

### Hipk induces melanotic masses in the hemocytes

**Fig. 2.**
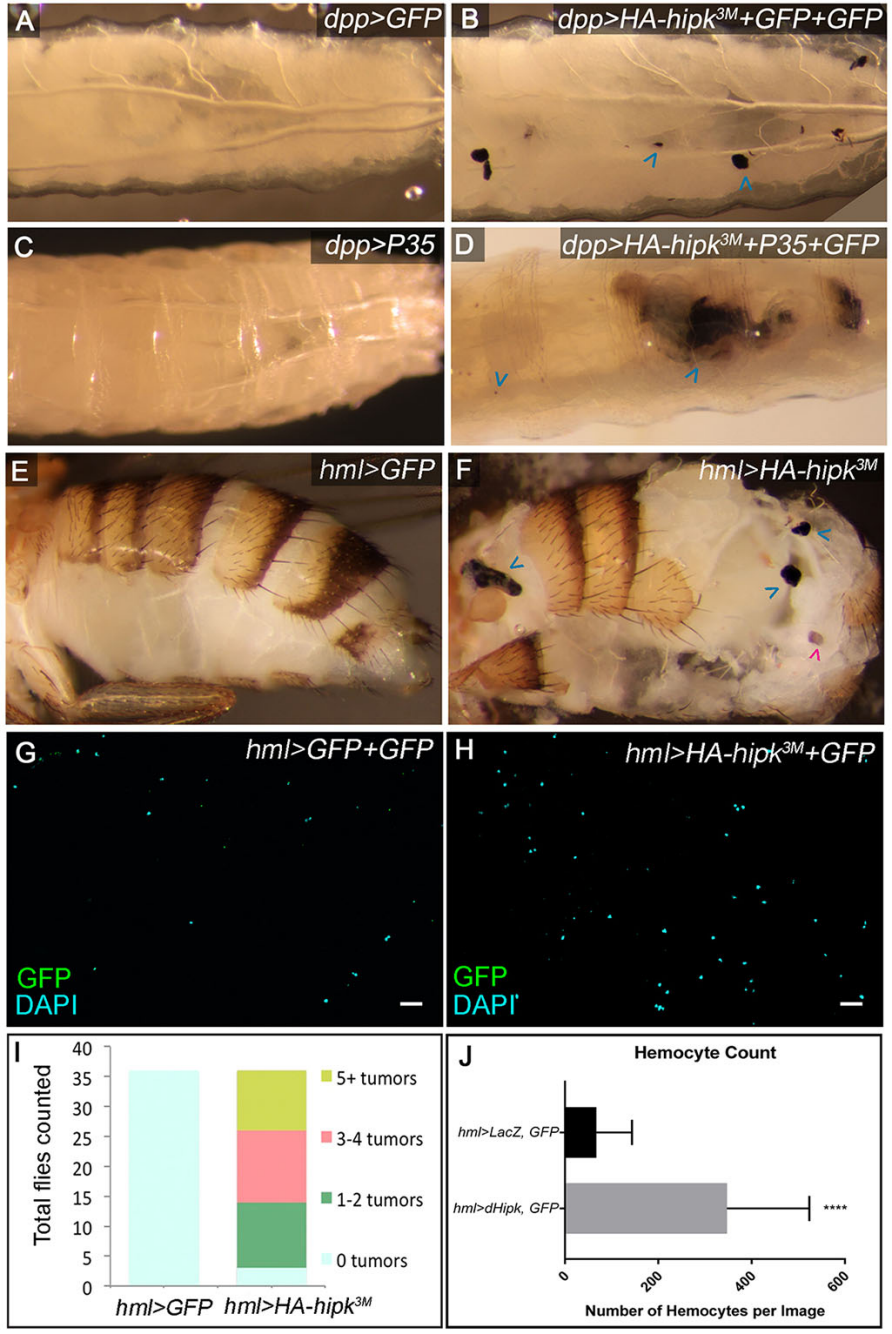
**Hipk induces hemocyte-derived melanotic tumors.** (A) A control *dpp>GFP* third instar larva. (B) Stationary melanized masses are observed in 65% of *dpp>HA-hipk^3M^+GFP+GFP* larvae (blue arrowheads; *n*=40). (C) *dpp>P35* larvae show no tumors. (D) The masses persist when apoptotic cell death is inhibited in *dpp>HA-hipk^3M^+P35+GFP* larvae. (E) The abdomen of a control *hml>GFP* fly. (F) Melanized tumors are present in *hml>HA-hipk^3M^* flies (blue arrowheads). Spermathecae were not counted (magenta arrowhead). Smears of total hemolymph collected from (G) *hml>2xGFP* and (H) *hml>HA-hipk^3M^+GFP* third instar larvae. (I) Quantification of the number of tumors scored from dissected abdomens of flies shown in (E) and (F), *n*=36 for both groups. (J) Quantification of mean number of hemocytes per defined sampling area counted from genotypes in (G) and (H). Each data point represents the mean of five cell counts from one larva, *hml>GFP+LacZ* (*n*=16 samples, *n*=80 cell counts) and *hml>HA-hipk^3M^+GFP* (*n*=16 samples, *n*=80 cell counts), ****P<0.0001.

In addition to overgrown discs, darkly pigmented stationary masses were present in both *dpp>HA-hipk^3M^+2xGFP* and *dpp>HA-hipk^3M^+P35+GFP* larvae grown at 29°C (Fig. 2B,D), whereas control larvae *dpp>GFP* (Fig. 2A) and *dpp>P35* (Fig. 2C) displayed none. The persistence of the masses upon *P35* coexpression suggests that they were not attributable to cell death. Moreover, *dpp>HA-hipk^3M^+P35+GFP* animals remained in the third larval stage for an extended period of time (beyond 10 days) and eventually died as larvae. Arrested development in tumor-ridden animals has been reported by others and is thought to be attributable to alterations in ecdysone regulation (Garelli et al., 2012; Parisi et al., 2014). We hypothesized that these tumors might arise because of expression of the *dpp-Gal4* driver in larval blood cells (Ayyaz et al., 2015; Clark et al., 2011).

Melanotic tumors arise as a result of over-amplification and melanization of hemocytes, which are fly hematopoietic cells (Hanratty and Ryerse, 1981). Therefore, we next tested directly whether *hipk* could cause tumors when overexpressed in the circulating hemocytes and lymph gland using *hemolectin-GAL4* (*hml-GAL4*) (Sinenko and Mathey-Prevot, 2004). Of the *hml>HA-hipk^3M^* flies, 91.7% exhibited at least one clearly visible melanotic tumor, with the average being three or four tumors (Fig. 2F,I), compared with 0% of *hml>GFP* flies (Fig. 2E,I). To test whether Hipk increased the number of circulating hemocytes, we isolated the total hemolymph from third instar larvae. The mean number of hemocytes in each *hml>HA-hipk^3M^+GFP* sampling area (see Materials and methods) was 348, compared with 67 per *hml>GFP+GFP* sampling area (Fig. 2G,H,J). These data suggest that the abdominal tumors induced by Hipk are derived from hyperproliferating hemocytes.

### Hipk induces cell invasiveness

Valuable methods have been developed that allow one to assay for invasive behavior using the GAL4-UAS system (Ferres-Marco et al., 2006; Herranz et al., 2014; Pallavi et al., 2012). In the wing disc, *dpp* is expressed in the anterior-most cells of the anterior-posterior (A/P) boundary. Thus, in *dpp>GFP* discs, a sharp border of GFP-expressing and non-GFP-expressing cells is produced (Fig. 1B). In *dpp>HA-hipk^3M^+GFP* discs, multiple isolated GFP^+^ cells were found outside the *dpp* domain, suggesting that cells migrated away from their original location in the disc (Fig. 1E).

**Fig. 3.**
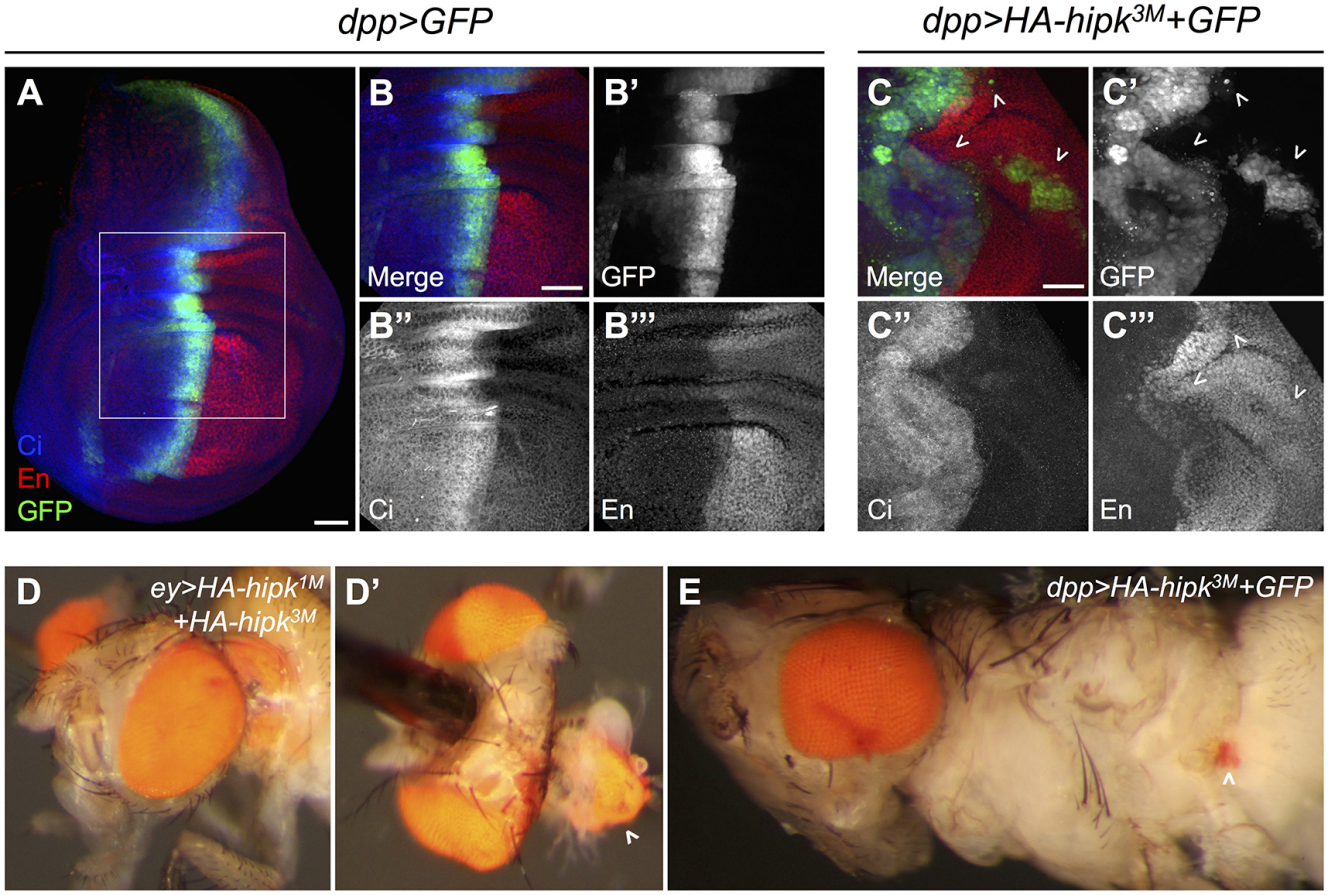
**Hipk induces cell spreading.** (A,B) A *dpp>GFP* wing disc stained for GFP, Ci and En. (C) In *dpp>HA-hipk^3M^+GFP* discs, anterior fated cells are found within the posterior wing compartment (arrowheads). (D) An ectopic eye seen within the thorax of an *ey>HA-hipk^1M^+HA-hipk^3M^* fly (frequency 1%, *n*=100). (D′) Dissection reveals the size of the ectopic eye (arrowhead). (E) A smaller ectopic eye is seen in the abdominal region of a *dpp>HA-hipk^3M^+GFP* fly (arrowhead; frequency 2%, *n*=100). Scale bars: 50 μm.

To provide further evidence of cell spreading, *dpp>HA-hipk^3M^+GFP* wing discs were co-stained for the anterior marker Cubitus interruptus (Ci) and for the posterior marker Engrailed (En) (Fig. 3A-C). In normal conditions, the A/P boundary is well defined, in which the Dpp domain (marked by GFP) is restricted within the anterior compartment (Fig. 3A,B). However, in discs with elevated *hipk*, GFP^+^ cells originating from the anterior compartment were found in the posterior domain as isolated clusters of cells (Fig. 3C′). On rare occasions, GFP^+^ clusters simultaneously expressed Ci and En, suggesting that these cells have either lost their ability to interpret A/P positional cues from the tissue or are in a period of fate transition (Fig. S3A). We also found that individual GFP^+^ cells move from the central Dpp domain towards both anterior and posterior parts of the discs (Fig. 1E).

Another phenotype associated with metastatic behavior in *Drosophila* is the migration of retinal tissue into the body of the fly (Ferres-Marco et al., 2006; Pallavi et al., 2012). When *hipk* was expressed in the eye disc using *eyeless-GAL4* (*ey-GAL4*), a large cluster of pigmented retinal cells was observed in the thorax of the adult fly (Fig. 3D). The endogenous eyes were fully intact, which suggests this was not likely to have been a disc eversion defect, but rather a metastatic event where retinal tissue migrated away from the eye disc and lodged into the thorax. This phenotype also occurred in *dpp>HA-hipk^3M^+GFP* flies, in which ectopic pigmented eye cells can occasionally be observed in the abdomen (Fig. 3E), indicating that Hipk-expressing eye disc cells can migrate within the body and proliferate.

The data presented thus far suggest that elevating *hipk* promotes proliferation, cell migration and, possibly, metastatic behavior. To test for this, we used live imaging and witnessed cell extrusion in real time. In *dpp>HA-hipk^1M^+HA-hipk^3M^* eye discs, cells proliferated at a high rate, and multiple cells could be seen extruding from the disc into the culture medium (Fig. S3D-F). Furthermore, the extruded cells continued to proliferate after leaving the disc. Within 60 min of imaging this particular disc, 12 cells left the disc into the culture media (Fig. S3F,G) and some continued amplifying to reach a final cell count of 21 over the subsequent 60 min (Fig. S3H-J). We did not observe any such occurrence in control discs (Fig. S3B,C). Together, these data suggest that cells with elevated Hipk can gain the potential to travel away from their original location in the epithelium.

### Hipk alters the integrity of the basement membrane and induces EMT

**Fig. 4.**
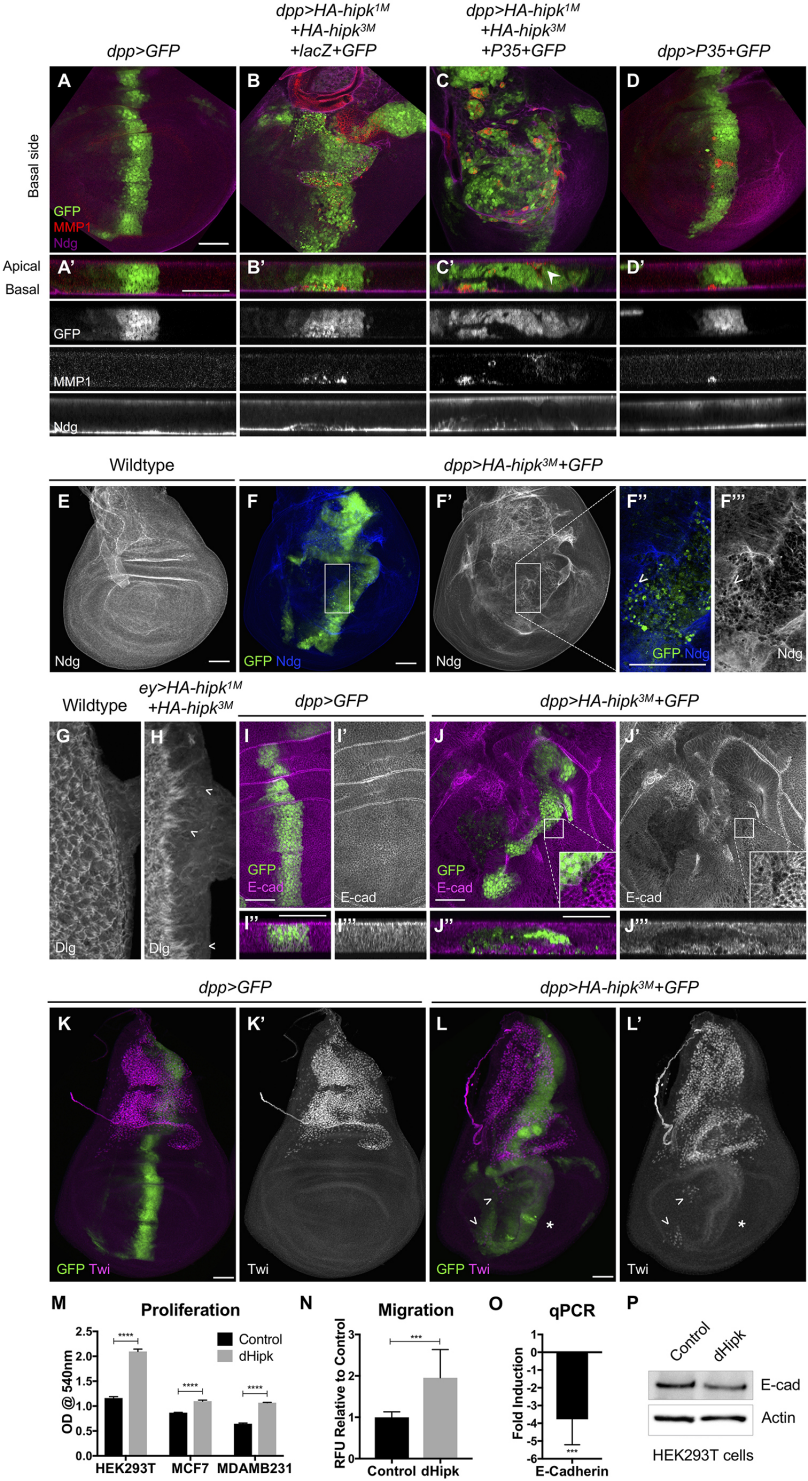
**Hipk alters epithelial integrity and induces EMT.** (A,A′) A control wing disc (A) and a cross-section of the central region of the disc (A′) stained for GFP, Mmp1 and Ndg. (B,B′) *dpp>HA-hipk^1M^+HA-hipk^3M^+LacZ+GFP* stained to detect GFP, Mmp1 and Ndg. (C,C′) *dpp>HA-hipk^1M^+HA-hipk^3M^+P35+GFP* stained to detect GFP, Mmp1 and Ndg. (D,D′) *dpp*>*P35+GFP* stained to detect GFP, MMP1 and Ndg. (E) Ndg is expressed in a uniform pattern along the basement membrane. (F-F‴) Gaps in Ndg expression are present in *dpp>HA-hipk^3M^+GFP* discs (F′), and higher resolution (F″,F‴) shows that the location of *HA*-*hipk^3M^+GFP*-expressing cells coincides with regions where Ndg is perturbed (arrowheads). (G) Wild-type eye disc stained for Dlg to reveal cell membranes. (H) *ey>HA-hipk^1M^+HA-hipk^3M^* eye disc stained for Dlg showing defects in Dlg stain and apparent cell extensions towards posterior margin of disc (chevrons). (I,J) Cross-sections of *dpp>GFP* (I) and *dpp>HA-hipk^3M^+GFP*-expressing cells (J) in the center of the wing pouch, stained for E-cad. (K) Twi is expressed in the adepithelial myoblasts, located in the notum region of the wing disc. (L) Twi-positive mesenchymal cells are present in the wing pouch region of *dpp>HA-hipk^3M^+GFP* discs (arrowheads), and Twi is induced in a swathe of cells along the *dpp* domain (asterisk). Scale bars: 50 μm. (M) *Drosophila* Hipk (dHipk) promotes significant proliferation of three cell lines. dHipk-transfected HEK293T cells (mean=2.097, s.e.m.=0.02803) display increased proliferation compared with empty vector transfected (mean=1.161, s.e.m.=0.01619) conditions; *t*(4)=28.92, ****P<0.0001. dHipk-transfected breast adenocarcinoma MCF7 cells (mean=1.098, s.e.m.=0.01217) display increased proliferation compared with empty vector transfected (mean=0.8671, s.e.m.=0.0035) conditions; *t*(4)=18.25, ****P<0.0001. dHipk-transfected breast adenocarcinoma MDA-MB-231 cells (mean=1.067, s.e.m.=0.0037) display increased proliferation compared with empty vector transfected (mean=0.6457, s.e.m.=0.0074) conditions; *t*(4)=50.83, ****P<0.0001. (N) dHipk-transfected MDA-MB-231 cells (mean=1.953, s.e.m.=0.2277) demonstrated significantly increased migration compared with empty vector transfected (mean=0.9999, s.e.m.=0.0375) conditions; *t*(19)=4.759, ***P=0.0001. (O) qRT-PCR was used to quantify the expression of the human E-cadherin gene (*CDH1*) after transfection of MDA-MB-231 cells with dHipk [*t*(2)=34.86, ***P=0.0008]. (P) Western blot analysis of E-cad expression in HEK293T cells after dHipk transfection.

During metastasis, cells extrude from the main epithelium through various mechanisms, including degradation of the basement membrane by matrix metalloproteinases, such as Mmp1 (Beaucher et al., 2007; Page-McCaw et al., 2003; Srivastava et al., 2007). Expression of Hipk using either *dpp-Gal4* or flip-out misexpression clones leads to elevated Mmp1 expression in a cell autonomous manner (Fig. 4A,B; Fig. S2B). Hipk was previously shown to induce Mmp1 expression, but only when the *smt3* gene encoding Small ubiquitin-related modifier (SUMO; Smt3) was simultaneously knocked down (Huang et al., 2011). Huang et al. (2011) suggested that in the absence of *smt3* Hipk translocates to the cytoplasm and induces JNK and its target, Mmp1. In our experimental context, when HA-*hipk^3M^* and *GFP* were expressed by *dpp-GAL4* at 29°C, Hipk was largely nuclear (Fig. S4A), suggesting that Mmp1 induction in our assay is probably attributable to another mechanism.

We examined the basement membrane by staining wing imaginal discs for Nidogen (Ndg), an extracellular matrix component (Fig. 4A-F; Wolfstetter et al., 2009). In *dpp>HA-hipk^3M^+GFP* discs, disruptions in the Ndg pattern were observed in sections of wing discs (Fig. 4B) and when the basal surface was examined en face (Fig. 4F). Specifically, the location of *HA-hipk^3M^+GFP*^+^ cells and expression of Mmp1 coincided with disruptions in Ndg (Fig. 4B). GFP^+^ Hipk-expressing cells appeared to be extruding through holes in the basement membrane (Fig. 4F). Cross-sections of discs also revealed that *hipk-*expressing cells appeared to be intercalated into the basement membrane (Fig. S4K), which is never seen in wild-type discs (Fig. S4I). Consistent with disruptions in Ndg, elevated *hipk* produced inconsistencies in Collagen IV (Viking, Vkg) in the wing disc (Fig. S4B,C). These data suggest that Hipk promotes Mmp1-mediated degradation of the basement membrane.

Coexpression of P35 with Hipk caused greater abnormalities in disc morphology, with multiple folds and cell layers, owing to the blocking of cell death (Fig. 4C). In this context, GFP^+^ cells were observed breaking through the disc surface at both the apical and basal surfaces (Fig. 4C′), suggestive of active migration processes rather than cell death being the mechanism driving cell migration and spreading. Cell autonomous alterations in Mmp1 and Ndg (Fig. 4C) were seen in disc sections, and elevated Mmp1 was seen within protrusions of *dpp>HA-hipk^3M^+P35+GFP* eye discs (Fig. S4E″). P35 expression alone is also capable of inducing Mmp1, but no defects in Ndg integrity were observed (Fig. 4D′; Rudrapatna et al., 2013).

We also observed abnormal cell behavior after staining eye discs to detect Dlg and Elav to reveal tissue architecture. In a wild-type disc, the posterior margin of the disc displays a tight boundary of photoreceptor cells as detected by Dlg (Fig. 4G) and Elav (Fig. S4F). In *ey>HA-hipk^1M^+HA-hipk^3M^* discs, the cell morphology is altered, and jagged cell extensions can be seen protruding towards the posterior margin using the Dlg antibody stain (Fig. 4H). Overall, altered integrity of the posterior margin is also seen when staining for Elav (Fig. S4G).

EMT occurs naturally in development (Kiger et al., 2007), but it can also be induced inappropriately during tumorigenesis. Characteristics of EMT are increased expression of Mmp1, mesenchymal markers like Twist (Twi) and Snail, and downregulation of E-cadherin (E-cad). In *dpp>HA-hipk^3M^+GFP* discs grown at 29°C, levels of E-cad are reduced in a cell autonomous fashion (Fig. 4J). Twist is normally expressed within mesenchymal cells found within the notum region of the wing disc (Herranz et al., 2014; Fig. 4K). When *hipk* was overexpressed, Twi expression was mildly induced along the *dpp* domain, and multiple cells within the wing pouch displayed ectopic expression of Twi (Fig. 4L). Others have shown that Hipk promotes epithelial remodeling of the pupal wing through an EMT-like mechanism (Kiger et al., 2007; Link et al., 2007).

### Hipk can induce proliferation and cell migration in vertebrate cells

To determine whether the properties of *Drosophila* Hipk are conserved in a different context, we examined the effects of transient transfection of *pCMV-HA-dHipk* into a number of human cell lines. We first assayed proliferation induced by Hipk in HEK293T, MCF7 and MDA-MB-231 human cell lines. After transient transfection, we used the MTT assay to measure cell proliferation and found that Hipk significantly stimulated cell proliferation in all three cell lines (Fig. 4M; Table S4). Using a cell migration assay, we found that transfection of *Drosophila* Hipk caused MDA-MB-231 cells to exhibit a twofold increase in migration relative to control cells (Fig. 4N; Table S3). One crucial aspect of EMT is the downregulation of E-cad expression. In MDA-MB-231 cells transfected with Hipk, E-cad levels were reduced 3.5-fold compared with levels found in control transfected cells (Fig. 4O). Further evidence for decreased E-cad is seen after western blotting of E-cad from HEK293T cells transfected with dHipk (Fig. 4P; Table S2). These observations support our observations from *Drosophila* tissues that Hipk is a potent factor that can promote proliferation and EMT in different contexts.

### Hipk-induced phenotypes cannot be attributed to a single targeted pathway

**Fig. 5.**
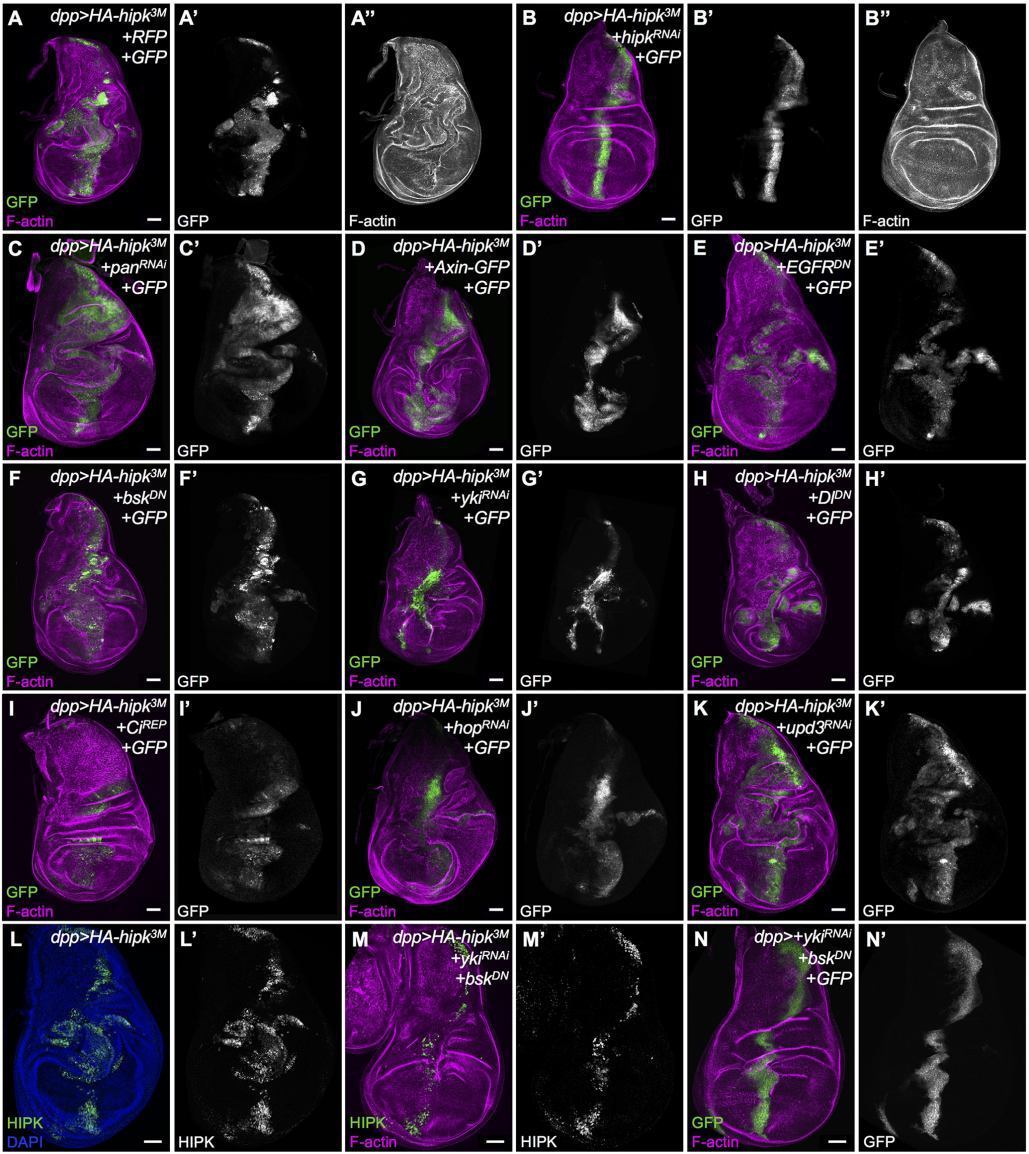
**Loss of individual signaling pathway components cannot suppress the Hipk overexpression phenotype.** We assessed the ability of knockdown of the activity of individual pathways to suppress phenotypes induced by overexpressed Hipk, by F-actin staining (magenta) to reveal morphology and GFP (green, white) to indicate cells in which genotypes were manipulated. (A,B) As proof of concept, *hipk^RNAi^* (B) suppressed effects seen in *dpp>HA-hipk^3M^* wing discs (A). The following pathways were targeted with the indicated transgenes: Wg, using (C) *UAS-pan^RNAi^* [dTCF] and (D) *UAS-Axin-GFP*; Egfr, using (E) *UAS-Egfr^DN^*; JNK, using (F) *UAS-bsk^DN^*; Hippo, using (G) *UAS-yki^RNAi^*; Notch, using (H) *UAS-Dl^DN^*; Hedgehog, using (I) *UAS-Ci^Rep^*; JAK/STAT using (J) *UAS-hop^RNAi^* and (K) *UAS-upd3^RNAi^*. (L) *dpp>HA-hipk^3M^* wing disc stained for nuclei (DAPI) and Hipk. (M) Expression of *dpp>HA-hipk^3M^*+*UAS-yki^RNAi^*+*UAS-bsk^DN^* stained to reveal Hipk and F-actin. (N) *dpp>UAS-yki^RNAi^*+*UAS-bsk^DN^* stained to reveal GFP and F-actin. All crosses were done at 29°C. Scale bars: 50 μm.

To investigate genetically the mechanism underlying the ability of Hipk to induce cell spreading, proliferation and migration, we assessed the effects of disruptions of individual pathways on the *dpp>HA-hipk^3M^* phenotype in wing discs by assaying the extent of cell proliferation and migration from the *dpp* expression domain (Fig. 5A). We chose pathways that were previously shown to be promoted by Hipk in various contexts, in addition to conserved tumor pathways. We evaluated the effects on proliferation and migration of the transgenes individually using *dpp-Gal4* (Fig. S5). We also validated that each transgene was effective by assaying targets or downstream events specific to each pathway (Fig. S6). To test whether the Hipk phenotype could be reverted, we first used RNAi against *hipk* and found that it completely rescued the abnormalities seen in *dpp>HA-hipk^3M^* discs (Fig. 5B). The proliferative and invasive effects caused by each transgene and their influence on the Hipk-induced phenotype were quantified by measuring the GFP^+^ cell area relative to total disc area (Fig. S7A; Table S5), and by assigning a ‘relative degree of invasiveness’ score to each disc (Fig. S7B; Table S8).

The Wg pathway was inhibited through either knockdown of *pangolin/TCF* (Fig. 5C) or expression of the negative regulator Axin (Fig. 5D). We noticed that *hipk-*expressing discs with loss of *pan* (*TCF*) still displayed the invasive phenotype and even some overgrowth in the notum region. Likewise, expression of Axin failed to suppress the *dpp>HA-hipk^3M^* phenotype. Wing discs coexpressing dominant negative Epidermal growth factor receptor (Egfr^DN^; Fig. 5E) or dominant negative *basket* (bsk^DN^, *Drosophila* JNK; Fig. 5F) with *hipk* were phenotypically indistinguishable from discs expressing *hipk* alone (*dpp>HA-hipk^3M^*). Knockdown of *yki* could reduce the overgrowth effect to some degree, consistent with the effect of Hipk on Hippo signaling, but the discs still showed ectopic cell migration (Fig. 5G,G′). Expression of dominant negative Delta (Dl^DN^; Fig. 5H) did not appreciably modify the Hipk overexpression phenotype. Interestingly, after inhibition of the Hedgehog pathway through expression of the repressor form of Ci (Ci^Rep^; Fig. 5I), the cell spreading phenotype seemed suppressed and the discs displayed only a broad Dpp domain. We also noticed relatively weak GFP expression in the discs, which is most likely to be attributable to the repression of *dpp-Gal4* expression, because Hh controls *dpp* transcription (Basler and Struhl, 1994). Reduction of JAK/STAT signaling through knockdown of *hopscotch* (*hop*; *Drosophila* JAK; Fig. 5J) showed mild reduction of proliferation, whereas knockdown of one of the unpaired ligands, Upd3, appeared to increase proliferation slightly (Fig. 5K). Together, our genetic data show that interfering with individual signaling pathways using expression of RNAi or dominant negative forms of the corresponding key effectors could not effectively suppress Hipk-induced cell proliferation and spreading.

To test whether the effects are attributable to multiple pathways, we simultaneously interfered with the activity of Yki and Bsk by expressing *yki* RNAi with bsk^DN^ in a *dpp>HA-hipk^3M^* background (Fig. 5M). Although expression of Hipk induces overproliferation of the *dpp*-expressing cells, which can be seen by staining for Hipk in *dpp>HA-hipk^3M^*, after inhibition of Yki and Bsk, the number of Hipk-expressing cells in the *dpp* domain is drastically reduced, and no cell spreading is observed. Expression of *dpp>yki*^RNAi^ with *bsk^DN^* alone had a mild effect on the number of GFP^+^ cells in the *dpp* stripe (Fig. 5N).

### Increasing the activity of individual signaling pathways does not phenocopy Hipk-induced phenotypes

**Fig. 6.**
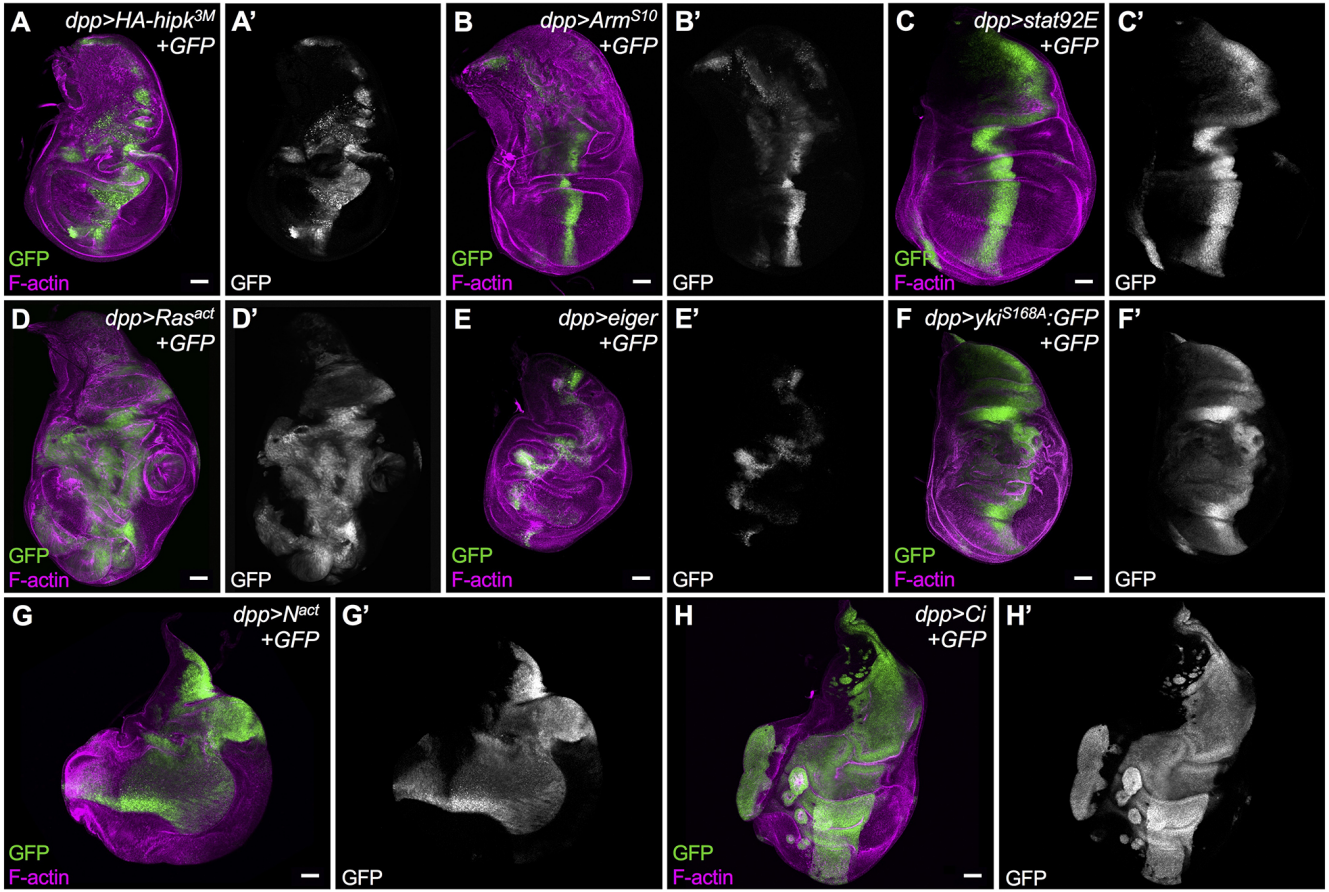
**Overexpression of individual signaling pathway components does not phenocopy the cell-spreading phenotype induced by elevated Hipk.** (A) A third instar wing imaginal disc with *HA-hipk^3M^+GFP* expressed along the *dpp* domain serves as the baseline phenotype/control disc. Individual pathway activators were expressed using *dpp-Gal4, UAS-GFP*, namely: (B) *UAS-Arm^S10^*, (C) *UAS-Stat92E*, (D) *UAS-Ras^act^*, (E) *UAS-eiger*, (F) constitutively active *UAS-yki^S168A^*, (G) *UAS-N^act^* and (H) *UAS-Ci*. Discs were stained for F-actin (magenta) to reveal tissue morphology and for GFP (green, white) to mark cells in which transgenes were ectopically expressed using *dpp-Gal4*. Scale bars: 50 μm. All crosses were done at 29°C.

We next examined whether hyperactivation of pathways that are promoted by Hipk, or that are involved in growth and proliferation, can induce similar phenotypes to those caused by overexpression of Hipk (Fig. 6A). This might reveal whether there are certain pathways that play a more dominant role in propagating the Hipk signal. We used UAS-controlled expression of wild type or constitutively active pathway members (Table S1). Wing discs expressing degradation-resistant Arm^s10^ (β-catenin) to promote Wg signaling (Fig. 6B) displayed ectopic wing pouch-like structure in the notum, yet the Dpp stripe appeared relatively normal. Overexpression of Stat92E to elevate JAK/STAT signaling (Fig. 6C) led to oversized discs. Wing discs expressing oncogenic Ras to promote Ras/Erk signaling showed robust overgrowths (Fig. 6D). Stimulation of the JNK pathway using *eiger* expression primarily caused invasive phenotypes but had little effect on proliferation (Fig. 6E). Inactivation of Hippo signaling by expression of constitutively active Yki (Yki^S168A^) led to widening of the Dpp domain, and smooth, curved edges along the domain (Fig. 6F). Activated Notch signaling (N^act^; Fig. 6G) and ectopic Ci (Fig. 6H), which promotes Hh, both induced very dramatic and unique cellular effects. *dpp>Notch^act^* led to phenotypes in the wing disc similar to those previously seen with expression of *dpp>Dl* (Ferres-Marco et al., 2006). Overall, this assay reveals that activation of different pathways leads to distinct effects on proliferation. These results suggest that Hipk-induced phenotypes are likely to arise as a cumulative effect of stimulating the activity of multiple pathways, because no single pathway can phenocopy the behavior of cells in discs with elevated Hipk in the *dpp* domain. The proliferative and invasive effects caused by each transgene were quantified by measuring the GFP^+^ cell area relative to total disc area (Fig. S7C; Table S6), and by assigning a ‘relative degree of invasiveness’ score to each disc (Fig. S7D; Table S9).

### Hipk overexpression synergistically enhances other tumor models

**Fig. 7.**
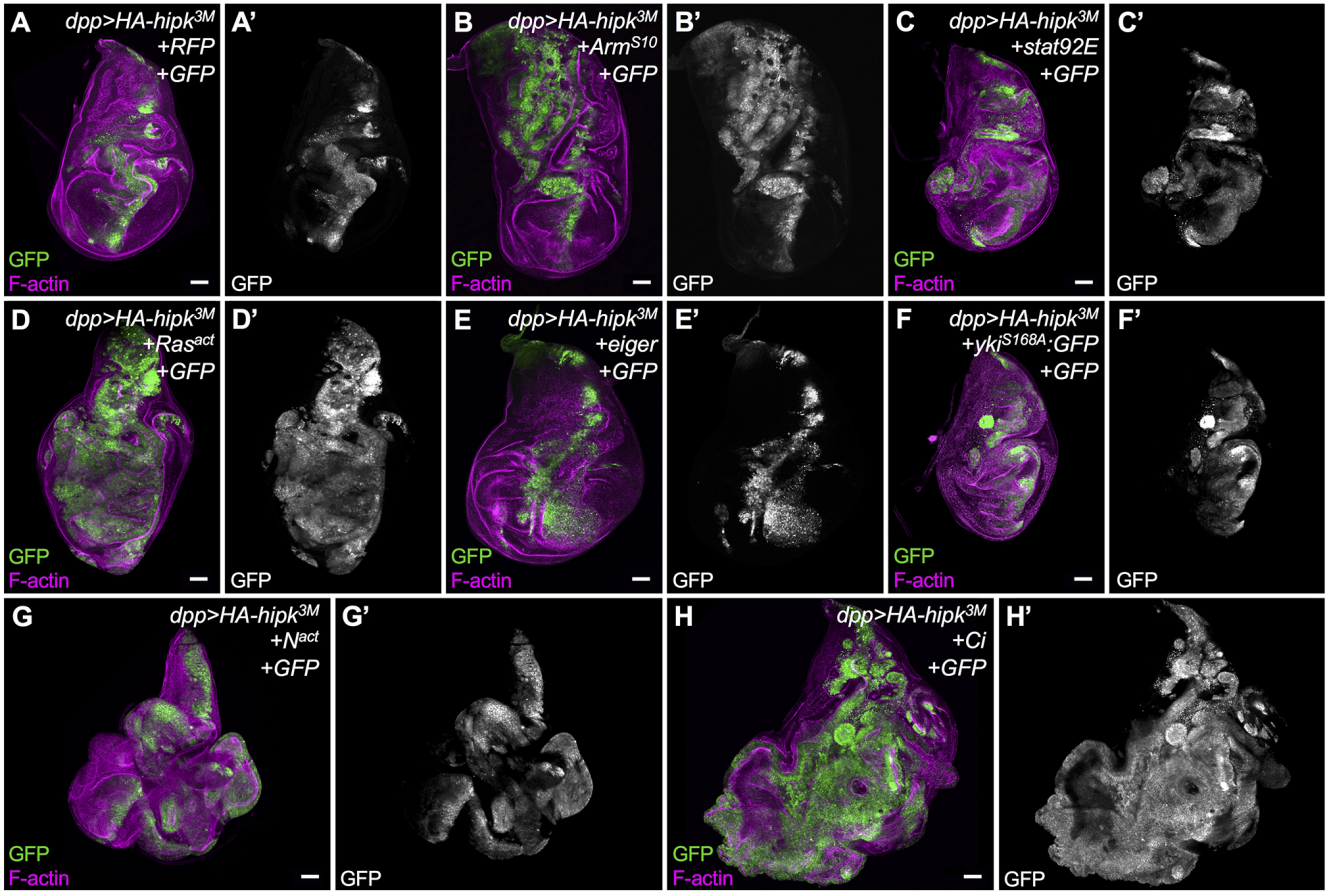
**Hipk overexpression synergistically enhances other tumor models.** Individual pathway activators were expressed by crossing to *dpp-Gal4, UAS-GFP; UAS-HA-hipk^3M^/TM6B*, namely: (A) Gal4 titration control crossed to *UAS-RFP*, (B) *UAS-Arm^S10^*, (C) *UAS-Stat92E*, (D) *UAS-Ras^act^*, (E) *UAS-eiger*, (F) constitutively active *UAS-yki^S168A^*, (G) *UAS-N^act^* and (H) *UAS-Ci*. Discs were stained for F-actin (magenta) to reveal tissue morphology and for GFP (green, white) to mark cells in which transgenes were ectopically expressed using *dpp-Gal4*. Scale bars: 50 μm. All crosses were done at 29°C.

Finally, we assessed whether Hipk expression could synergize with other sensitized tumor models in *Drosophila* wing discs. We coexpressed Hipk with the same gain of function mutants used in the previous section (Table S1) and assayed proliferation and cell migration. The phenotype of *dpp>Arm^s10^* alone was enhanced upon coexpressing *HA-hipk^3M^*; notably, the effect was much more pronounced in the notum region of the disc (Fig. 7B). Coexpression of *stat92E* and *hipk* resulted in invasive phenotypes (Fig. 7C), whereas discs expressing *stat92E* alone did not (Fig. 6C). In stark contrast to the phenotype in *eiger*-expressing discs (Fig. 6E), Hipk cooperated with Eiger to cause a significant increase in migrating cells (Fig. 7E). Despite being smaller than *dpp>yki* discs, *dpp>hipk+yki* discs acquired noticeable cell spreading properties (Fig. 7F). Ras^act^ (Fig. 7D) and Notch^act^ (Fig. 7G) both showed a strong synergistic effect with ectopic Hipk, compared with phenotypes seen with either one alone, shown in Fig. 6. The strongest synergy was seen with Ci (Fig. 7H). Of note, the effect with Ci alone was also the most dramatic in these assay conditions. The effects of all transgenes on Hipk-induced phenotypes were quantified by measuring the GFP^+^ cell area relative to total disc area (Fig. S7E; Table S7) and by quantifying the relative degree of invasiveness (Fig. S7F; Table S10). Thus, Hipk expression can synergize with several well-described *Drosophila* tumor and metastasis models, supporting its oncogenic properties.

## DISCUSSION

Accumulating evidence has strongly indicated that mammalian HIPKs are implicated in various diseases and cancers (reviewed by Blaquiere and Verheyen, 2017). However, whether HIPKs act as oncogenes or tumor suppressor genes remains ambiguous, possibly in part because of the genetic heterogeneity of different cancer types. In addition, comprehensive analyses of the four HIPK isoforms are lacking. Given the diverse expression patterns, distinct subcellular localization and potential functional redundancy of HIPK proteins, considerable efforts are needed to identify the roles of individual isoforms in each cell context, not to mention in unstressed or stressed conditions (for example, ultraviolet induction or hypoxia; Schmitz et al., 2014). In light of these complications, we decided to use *Drosophila*, a simple genetic model organism containing only one well-conserved Hipk, in most of our studies to unravel the roles of Hipk proteins in tumorigenesis.

Our work reveals that elevated expression of a single gene, *hipk*, in *Drosophila* tissues is sufficient to produce features of transformed tumors. We provide evidence that Hipk induces hyperplasia in imaginal discs and hemocytes, leading to massive tissue growth and melanotic tumor-like masses, respectively (Figs 1 and 2). Importantly, cells with elevated Hipk display protruding shapes and gain the potential to spread away from their primary site of origin (Figs 3 and 4). Furthermore, we provide evidence that Hipk induces basal invasion through mechanisms such as Mmp1-mediated degradation of the basement membrane and induction of EMT (Fig. 4). We also demonstrate that expression of *Drosophila* Hipk in the human aggressive breast cancer line MDA-MB-231 can potentiate proliferation, migratory behaviors and, by extension, EMT (Fig. 4).

We speculated that Hipk might trigger EMT in *Drosophila* tissues and vertebrate cells through conserved molecular mechanisms. Our studies uncover previously unrecognized functions of *Drosophila* Hipk in mediating metastasis. Our conclusions are in agreement with some studies reporting that human HIPK2 promotes EMT in renal fibrosis (Jin et al., 2012; Huang et al. 2015). HIPK2 expression has been shown to be remarkably upregulated in kidneys of patients with HIV-associated nephropathy, diabetic nephropathy and severe IgA nephropathy (Huang et al., 2015). Moreover, certain human cancers display elevated levels of HIPK2 within tumorous tissue (Al-Beiti and Lu, 2008; Deshmukh et al., 2008; Jacob et al., 2009). We infer that *Drosophila* Hipk mimics human HIPK2 in these fibrosis and tumor models. By contrast, another study found that in bladder cancer metastasis, downregulation of HIPK2 induced EMT and cell invasion (Tan et al., 2014). The cue for the switch of roles of HIPKs between EMT promotion and EMT suppression requires further investigation.

To elucidate the molecular mechanism by which Hipk can confer both proliferative and migratory properties on cells, we examined genetic interactions between Hipk and tumorigenic pathways that are known, or proposed, to be regulated by Hipk. First, we noticed that interfering with the activity of an individual signaling pathway is not sufficient to suppress both Hipk-mediated cell spreading and invasive phenotypes (Fig. 5). Second, stimulation of single pathways fails to recapitulate all the phenotypes induced by Hipk overexpression (Fig. 6). Of note, we do find that inhibition of individual pathways can suppress a subset of Hipk-induced phenotypes. For example, knockdown of Yki in a Hipk overexpression background inhibited cell proliferation, but did not have a strong effect on cell spreading. Conversely, inhibition of Hedgehog signaling did not affect proliferation, but appeared to reduce cell spreading. We were able to observe that the combined inactivation of Yki and Bsk was able to ameliorate most Hipk phenotypes. We propose that elevation of a single protein kinase, Hipk, even without accumulation of additional mutations, is likely to be potent enough to perturb multiple signaling pathways and, ultimately, have a cumulative effect of oversized, proliferative and protruding phenotypes. This mechanism, in effect, mimics tumor initiation attributable to multiple activating mutations in distinct pathways. Previously described *Drosophila* tumor models involve concomitant mutations that enhance proliferation, such as activated Ras, along with loss of cell polarity genes, such as *scribble*, to drive invasive behavior (Pagliarini and Xu, 2003).

Consistent with our proposed mechanism, HIPK2 is thought to mediate EMT by activation of EMT-promoting pathways, including TGFβ, Wnt and Notch (Huang et al. 2015). We believe that, in the future, profiling the transcriptome and the protein/protein and protein/DNA interactions in Hipk-expressing cells will give us an unbiased and thorough analysis of alterations of the signaling network upon Hipk overexpression.

The versatility of Hipk functions raises concerns regarding how we can block Hipk-induced phenotypes efficiently. Although inhibition of multiple downstream effectors of Hipk might be an option, we notice that impeding Hipk expression through *hipk-RNAi* can strongly reverse the overgrowth and cell spreading phenotypes (Fig. 5B). In line with our suggestion, a previous study proposed that exogenous overexpression of *miR-141*, which targets the 3′UTR of HIPK2, represented a potential approach to hinder HIPK2-mediated EMT (Jin et al., 2012). Given the large roles of post-translational modifications in Hipk protein turnover and localization (reviewed by Saul and Schmitz, 2013), we consider that mutations in other genes encoding Hipk regulators might also contribute to tumorigenesis even in the absence of *Hipk* gene mutations or changes in transcription levels of Hipk. Thus, revealing the regulation of Hipk activity is crucial to avoid Hipk-induced deleterious effects and to develop promising therapeutic interventions for Hipk-related disorders.

Lastly, we notice that Hipk is able to cooperate with other sensitized tumor models, probably in both additive and synergistic manners (Fig. 7). This implies that Hipk itself can elicit tumor-like transformations during the early phases of tumorigenesis. During the later phases, when multiple genetic alterations occur, Hipk might play an important role in accelerating tumor progression and metastasis. Future research would need to validate whether the human counterparts, HIPK1-HIPK4, can play roles in cancer initiation and progression in specific cancer types, and whether the functions of HIPK isoforms are redundant or disparate.

## MATERIALS AND METHODS

### Genetic crosses and fly stocks

Flies were raised on standard media, and *w^1118^* was used as wild type. A commonly used assay for proliferation and cell invasion is the use of *ey-Flp* to induce clones of tumor suppressors or expression of oncogenes in the eye-antennal disc (Pagliarini and Xu, 2003). We could not use this assay because of the inhibition of early eye specification mediated by Hipk (Blaquiere et al., 2014). All crosses were raised at 29°C to increase the effectiveness of GAL4-driven UAS constructs unless otherwise noted. All genetic interaction studies included controls for GAL4 titration through the use of benign UAS lines such as *UAS-GFP*, *UAS-RFP* or *UAS-lacZ* to match the UAS construct dose in experimental crosses. Fly strains used in this study were as follows: (1) *;;dpp-GAL4/TM6B* (Staehling-Hampton et al., 1994); (2) *;vkg-GFP;* (Flytrap); (3) *;UAS-HA-hipk^1M^*; (4) *;;UAS-HA-hipk^3M^* [both (3) and (4) are wild-type Hipk transgenes inserted on different chromosomes, which were previously reported as UAS-Hipk (II) and UAS-Hipk (III), respectively (Swarup and Verheyen, 2011)]; (5) *;;dpp-GAL4, UAS-HA-hipk^3M^/TM6B* [recombinant derived from stocks (1) and (4)]; (6) *;UAS-eGFP;* (BL#5431); (7) *;;UAS-eGFP* (BL#5430); (8) *;UAS-P35;* (BL#5072) (Hay et al., 1994); (9) *;hml-GAL4;* (BL#30139); (10) *;ey-GAL4;* (BL#5535); (11) *;;UAS-Axin-GFP* (BL#7225); (12) *UAS-Egfr^DN^* (dominant negative) with inserts on both II and III; (13) *UAS-bsk^DN^;;* (BL# 6409); (14) *;;UAS-yki^RNAi^* (BL#34067); (15) *;UAS-Dl^DN^;* (BL#26697); (16) *UAS-Ci^Rep^*; (17) *;UAS-arm^s10^;* generated in our laboratory (Mirkovic et al., 2011); (18) *;UAS-Stat92E;*; (19) *UAS-Ras^act^* (from H. Richardson); (20) *;;UAS-Eiger*; (21) *;UAS-yki^S168A^:GFP;*; (22) *;;UAS-N^act^*; and (23) *;UAS-Ci^5M^;.* Strains obtained from the Bloomington *Drosophila* Stock Center (Bloomington, IN, USA) have BL# stock numbers indicated. The following RNAi lines were primarily obtained from the Vienna *Drosophila* Resource Center (VDRC): (24) *;UAS-hipk^RNAi^;* (VDRC#108254); (25) ;*UAS-hop^RNAi^;* (VDRC#102830); (26) *UAS-upd^RNAi^*; (27) *;;UAS-pan^RNAi^* (TCF; VDRC#3014); (28) *y,w,hsflp[122]; sp/CyO; Act>CD2>GAL4,UAS-RFP/TM6B*; (29) *Dll-lacZ*; (30) *puc-LacZ*; (31) *10xStat92E-GFP* (BL#26197) (Bach et al., 2007); and (32) *MS1096-GAL4* (BL#8660).

### Antibodies and microscopy

Third instar imaginal discs were dissected and stained using standard protocols and, in most cases, we analyzed ≥20 discs per genotype. The following primary antibodies were used: mouse anti-Mmp1 (1:100: 3A6B4, 3B8D12, 5H7B11 DSHB; Rubin, G.M.), rat anti-Ci (1:20; 2A1 DSHB; Holmgren, R.), mouse anti-En (1:10; 4D9 DSHB; Goodman, C.), mouse anti-Dlg (1:100; 4F3 DSHB; Goodman, C.), mouse anti-HA (1:200; ABM), rabbit anti-Cas3 (1:100; 9661S; Cell Signaling), rabbit anti-Ndg [1:500; gift of Anne Hölz; (Wolfstetter et al., 2009)], rabbit anti-Twi [1:3000; gift of Maria Leptin (Roth et al., 1989)], mouse anti-beta-Galactosidase (1:50; 40-1a DSHB; Sanes, J.R.), rabbit anti-CycE (1:100; d-300; Santa Cruz), mouse anti-Wg (1:40; 4D4 DSHB; Cohen, S.M.), mouse anti-Cut (1:50; 2B10 DSHB; Rubin, G.M.), mouse anti-Ptc (1:40; Apa1 DSHB; Guerrero, I.). Rabbit anti-Hipk antibodies were generated in our laboratory and used at 1:200 dilution. The following secondary antibodies were obtained from Jackson ImmunoResearch: DyLight649 anti-rabbit, DyLight649 anti-rat, Cy3 anti-mouse and Cy3 anti-rabbit. Nuclei were detected by staining with DAPI, and F-actin was detected by staining with Rhodamine phalloidin (R-415; Molecular Probes). Immunofluorescent images were acquired using a Nikon Air laser-scanning confocal microscope. For live imaging, dissected eye discs were placed on a depression slide containing insect media and two larval brains. Discs were imaged using differential interference contrast microscopy (DIC) once per minute over 2 h (*n*=5 for each genotype). Whole larvae were mounted in Hoyer's medium, allowed to sit for 2 min, and imaged with a Canon Rebel T1i. Images were processed with Nikon Elements, Adobe Photoshop, Adobe Illustrator, ImageJ and Helicon Focus. For a subset of fluorescent images, channel colors were converted to accommodate color-blind viewers.

### Hemocyte counts

Before hemolymph collection, third instar larvae (*hml>GFP+LacZ* and *hml>HA-hipk^3M^+GFP*) grown at 29°C were washed thoroughly with 1× PBS, dried, and placed in glass dissection wells containing 10 μl of 1× PBS. The cuticle of single larvae was carefully punctured ventrally with forceps, and hemolymph was allowed to drain into the well for 30-60 s. The hemolymph was mixed with a pre-wetted pipet, and 1.5 µl of the hemolymph mixture was transferred to a poly-d-lysine-treated eight-well chamber-slide (BD Falcon CultureSlides; product #354108). Five 1.5 µl droplets were plated per larva, after which they were air dried. The dried samples were washed with 4% formaldehyde for 2 min, washed with PBS, and stained with DAPI. For each sample (*n*=16), five cell counts were performed from images taken at the center of each droplet at 200× magnification, and means of the five cell counts were plotted; values were subjected to Student's unpaired two-tailed *t*-test.

### Cell culture

MDA-MB-231 (ATCC; CRM-HTB-26), MCF7 (ATCC; HTB-22) and HEK293T (ATCC; CRL-3216) cell lines were grown in DMEM/F-12 (Dulbecco's Modified Eagle Medium/Nutrient Mixture F-12; Gibco; Cat. #: 11330032) with 10% fetal bovine serum (FBS).

### Cell transfection

MDA-MB-231 cells were transiently transfected with pCMV-myc empty vector (control) and pCMV-HA-dHipk vector using Lipofectoamine 3000 (Invitrogen) according to the manufacturer's instructions. Two micrograms of plasmid was used for each well in a six-well plate.

### Cell proliferation

Cell counts in transfected MDA-MB-231 cells were determined using an MTT proliferation assay using 3-(4,5-dimethylthiazol-2-yl)-2,5-diphenyltetrazolium bromide (Thermo Fisher, M6494). MDA-MB-231 cells were seeded into six-well plates (VWR, Cat# 10062-892) on day 1 to allow cells to attach, and transfections were performed on day 2. On day 3, 5000 cells were seeded into a 96-well plate, and on day 5, 10 μl 12 mM MTT stock solution was added into 100 μl medium in each well and incubated at 37°C for 3 h. Culture medium was then removed and 100 μl DMSO added to each well and incubated for 10 min at 37°C. We then read the optical density at 540 nm. Three replicates were performed for each condition in triplicate. Values were calculated as the mean±s.e.m. Significance between samples was assessed using Student's unpaired two-tailed *t*-tests. All raw data are provided in Table S4.

### Migration assay

MDA-MB-231cells were seeded at 80% confluence into six-well plates for 24 h and then transfected with pCMV-Myc empty vector or pCMV-HA-dHipk for 6 h, after which the medium was changed to starving medium (DMEM/F-12 without FBS) for 24 h. Then transfected cells were trypsinized (0.25% Trypsin-EDTA; Gibco) and counted using Trypan Blue, and 20,000 cells were suspended in 200 μl serum-free DMEM/F-12, seeded into the upper chamber of each insert (24-well insert, pore size of 8 μm; Greiner Bio-one). Eight-hundred microliters of DMED/F-12 containing 50% FBS was added to the bottom wells. After 24 h at 37°C, the culture medium was replaced with 450 μl serum-free medium plus 8 μm calcein-AM, incubated for 45 min at 37°C and then 500 μl trypsin was used to release the cells that had migrated through the membrane, incubating for 10 min. Two-hundred microliters of trypsin solution with detached migrated cells was transferred into a black flat-bottomed 96-well plate, and fluorescence was measured with an excitation wavelength of 485 nm and an emission wavelength of 520 nm. Three replicates were performed for each condition in triplicate. Values were calculated as the mean±s.e.m. Significance between samples was assessed using Student's unpaired two-tailed *t*-test. All raw data are provided in Table S3.

### RNA extraction and qPCR

Total RNA was isolated from cells using RNeasy Mini kits (Qiagen; 74101). First strand cDNA was synthesized from 0.5 μg RNA by PrimeScript First Strand cDNA Synthesis Kit (TaKaRa; 6110A). qPCR was performed using FastStart SYBR Green Master (Roche; 04673484001) on StepOne real-time PCR (ABI). *HPRT* was used as a housekeeping gene control. Primers used were as follows: *hHPRT* forward GCTATAAATTCTTTGCTGACCTGCTG; *hHPRT* reverse AATTACTTTTATGTCCCCTGTTGACTGG; *hE-Cad* (*CDH1*) forward GGACTTTGGCGTGGGCCAGG; and *hE-Cad* (*CDH1*) reverse CCCTGTCCAGCTCAGCCCGA. Relative fold levels were determined by the 
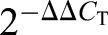
 method. Statistical significance was confirmed with Student's unpaired two-tailed *t*-test, with a theoretical mean set to one. All raw data are provided in Table S2.

### Western blot

Whole cell lysates were prepared with Cell lysis buffer (Cell Signaling Technology; #9803), supplemented with 1× Protease Inhibitors (Roche; #04693132001) and 1 mM PMSF before use. Protein lysates with 1× SDS sample buffer were subjected to 8% SDS-PAGE, followed by western blotting. The blots were detected by using the Pierce ECL Western Blotting Substrate (#32209). Images were captured with the use of FujiFilm LAS-4000 Chemi-luminescent Scanner. Rabbit anti-E-cadherin (1:1000; #3195; CST) and mouse beta-Actin (1:1000; G043; Abm) were used as primary antibodies. Anti-mouse and anti-rabbit HRP light-chain specific were used as secondary antibodies at 1:5000 (Jackson ImmunoResearch).

### Imaginal disc size measurements and invasiveness scoring

Experimental sets from Figs 5-7 were quantified for two parameters. First, the proliferative effects of each transgene were assessed by measuring the area of the GFP^+^ cells (driven by *dpp-GAL4*) and dividing it by the total disc area. Area measurements were taken in Image J from the .nd2 file for each disc. Measurements were calculated as ratios of the *dpp* stripe area to the total disc area (*dpp*/total; Fig. S7; Tables S5-S7). The difference in ratios was then quantified for Figs 5 and 7 using one-way ANOVA [Fig. S7-5, *F*(12,136)=41.88, *P*<0.0001; Fig. S7-7, *F*(7,81)=49.94, *P*<0.0001], with the Holm–Sidak post hoc test applied for multiple comparisons to 5A or 7A in Fig. S7-5 or Fig. S7-7, respectively. The scores for the Holm–Sidak tests are depicted in Fig. S7 as ‘ns’=not significant, *P<0.05, **P<0.01, ***P<0.001 and ****P<0.0001. Second, the invasive effects caused by each transgene were assessed by assigning a ‘relative degree of invasiveness’ score to each disc. We defined the ‘relative degree of invasiveness’ scores as follows: ‘None’=no cells found outside the normal *dpp>GFP* region; ‘Weak’=a few cells emerging from the *dpp>GFP* region, not only a widened GFP region attributable to proliferation; ‘Moderate’=extensions of cells that have traveled to edges of the hinge region; or ‘Strong’=all of the above and some solitary GFP masses found distinct from the main *dpp>GFP* region.

## Supplementary Material

Supplementary information
