# Supplementary material for: Homeodomain-interacting protein kinase promotes tumorigenesis and metastatic cell behavior
Source: Dis Model Mech. 2018 Jan 1;11(1):dmm031146. doi: 10.1242/dmm.031146 (PMC5818076; doi:10.1242/dmm.031146)
Supplement: Supplementary information [file dmm-11-031146-s1.pdf]

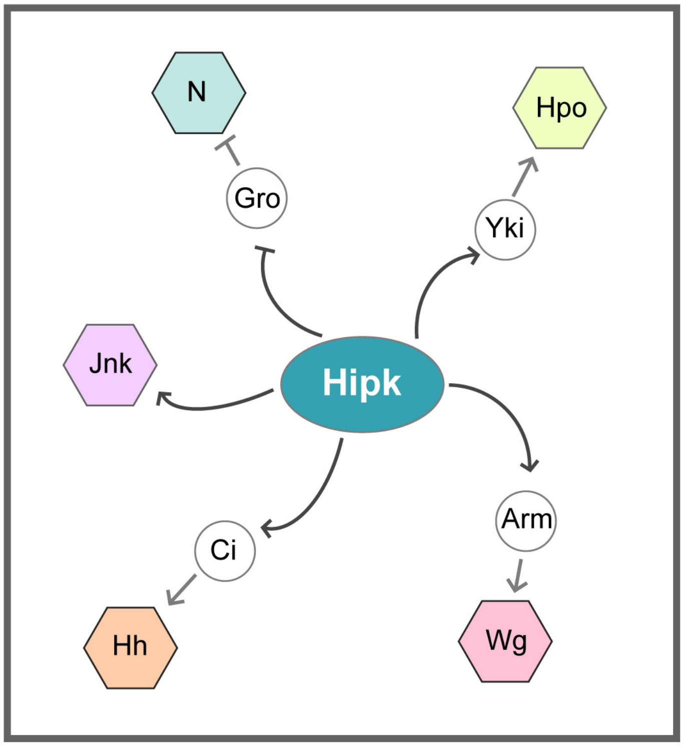

Figure S1. Hipk regulates numerous signaling pathways. A schematic diagram depicting Hipk's known relationships with many of the conserved signaling pathways.

Disease Models & Mechanisms • Supplementary information

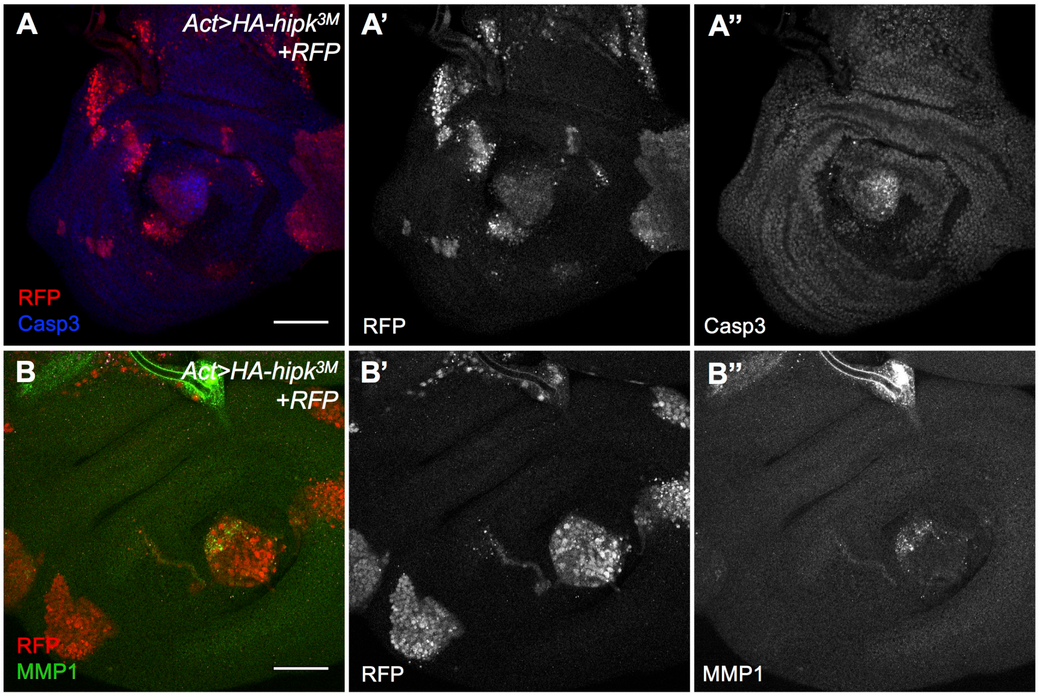

Figure S2: Hipk induces cell autonomous expression of Cleaved Caspase 3 and MMP1. Actin flp-out clones expressing *HA-hipk*^{3M} and marked with *UAS-RFP* were generated in wing imaginal discs. Anti-cleaved Caspase 3 antibody (shown in blue, A, A") and anti-MMP1 staining (shown in green, B, B") reveal low level cell autonomous induction.

Disease Models & Mechanisms • Supplementary information

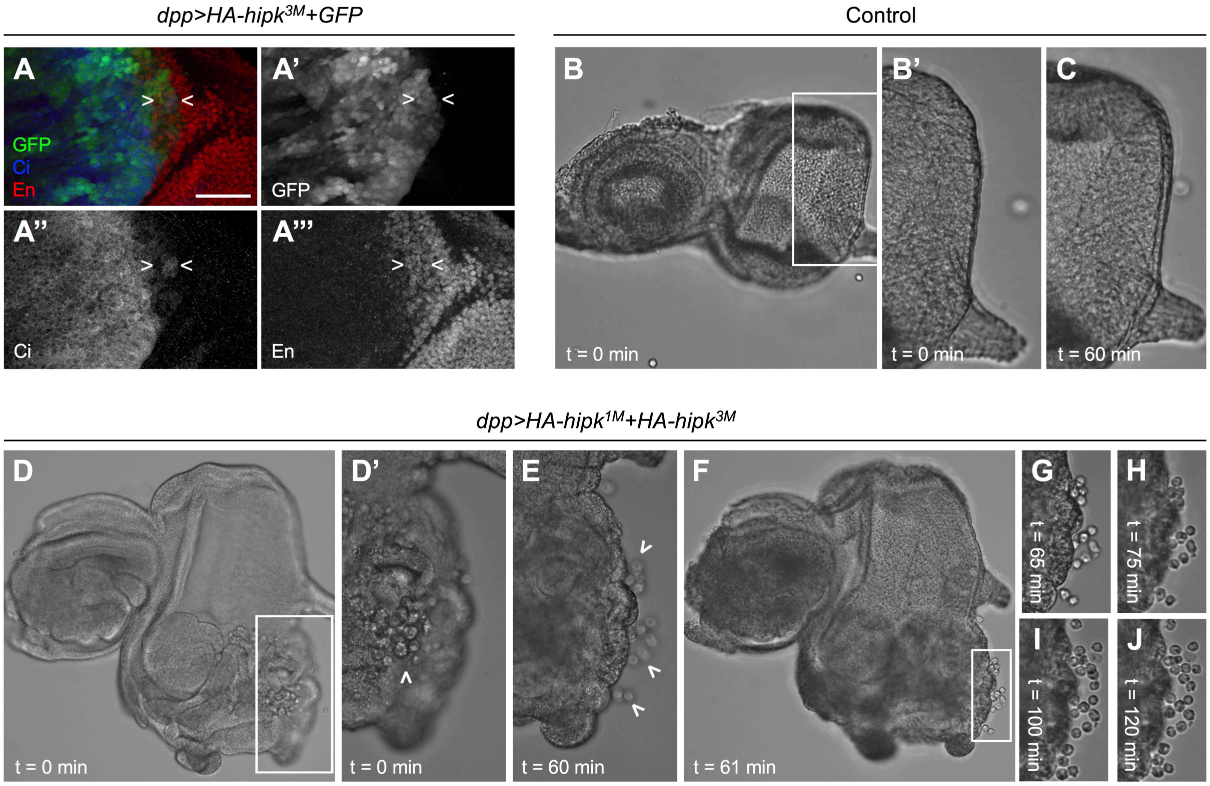

Figure S3: Hipk induces cell spreading. (A) A zoom-in of a *dpp>HA-hipk^{3M}+GFP* wing disc (similar to Fig. 3C) stained for Ci and En. Arrowheads mark a cluster of cells that simultaneously express GFP (**A**'), Ci (**A**''), and En (**A**'''). (B) A DIC image of a control eye disc. Live imaging stills of the zoomed-in control disc show that cells do not leave the disc after 60 mins (B', C). (D) At t = 0 mins a ventral overgrowth is present in the *dpp>HA-hipk^{1M}+HA-hipk^{3M}* eye disc. Zoomed-in images (D', E) reveal that cells leave the disc over a 60-min period (arrowheads). (F-J) At t = 65 mins, 12 extruded cells are next to the disc. Over the next 55 mins, those 12 cells amplify to reach a cell count of 21 (J). Boxed-in regions represent areas of corresponding zoom-ins. Scale bars equal 50μm.

Disease Models & Mechanisms • Supplementary information

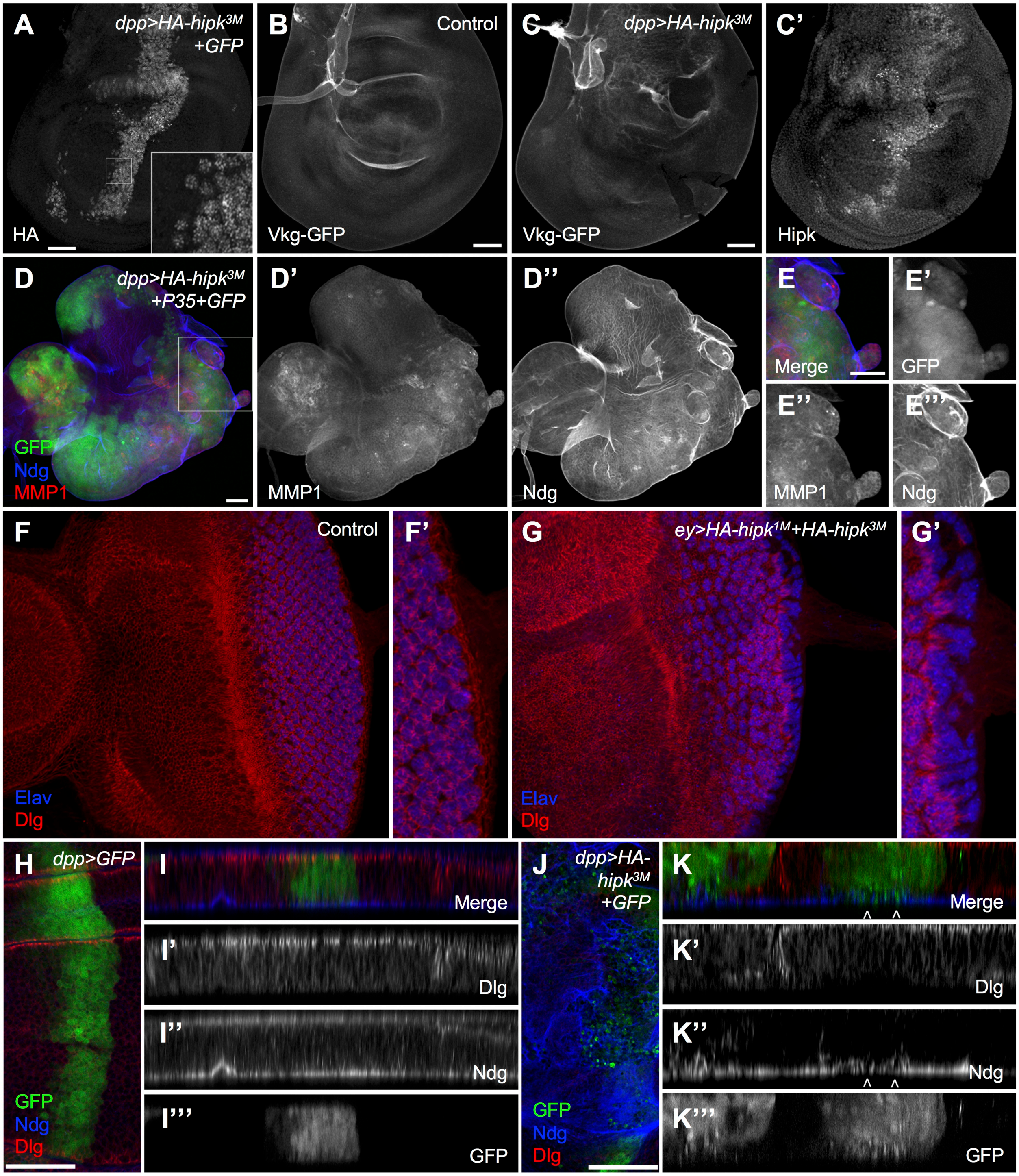

Disease Models & Mechanisms • Supplementary information

Figure S4: Hipk alters epithelial integrity.

(A) Anti-HA antibody reveals that *UAS-HA-hipk^{3M}* expression is nuclear (inset shows zoom-in). (B) A control disc shows the expression pattern of a basement membrane component Collagen IV (called Viking, Vkg, in Drosophila) using *Vkg-GFP.* (C) In *dpp>HA-hipk^{3M}* wing discs, inconsistencies in the normal Vkg pattern are present in the *dpp-Gal4* expression domain. (C') anti-Hipk stain in *dpp>HA-hipk^{3M}* reveals that effects on Vkg are cell autonmous. (D-D") When cell death is blocked in *dpp>HA-hipk^{3M}+P35+GFP* wing discs, MMP1 is dramatically up-regulated and Ndg is disrupted (compared to normal pattern seen in Fig. 4C). (E-E''') Zoom in on white box shown in D reveals high levels of MMP1 in GFP positive cells, as well as abnormal Ndg stain indicating disruptions in basement membrane. (F) Control eye disc stained for Elav (blue) and Dlg (red). (F') zoom of posterior region of eye disc. (G) ey>*HA-hipk^{1M}+HA-hipk^{3M}* disc stained for Elav to reveal photoreceptors (blue) and Dlg to highlight apical domains (red). (G') zoom of posterior region of eye disc. (H) *dpp>GFP* expressing cells in the center of the wing pouch, stained for Dlg to highlight apical domains and Ndg to reveal basement membrane, and GFP to show *dpp* expression domain. (I) Cross section of tissue shown in Fig. 4H. (J) A *dpp>HA-hipk^{3M}+GFP* disc stained for Dlg, Ndg and GFP. (K) A cross section of disc shown in Fig. 4J. Chevrons in K, K" reveal sites of intercalation of GFP positive cells with basement membrane. Scale bars equal 50μm.

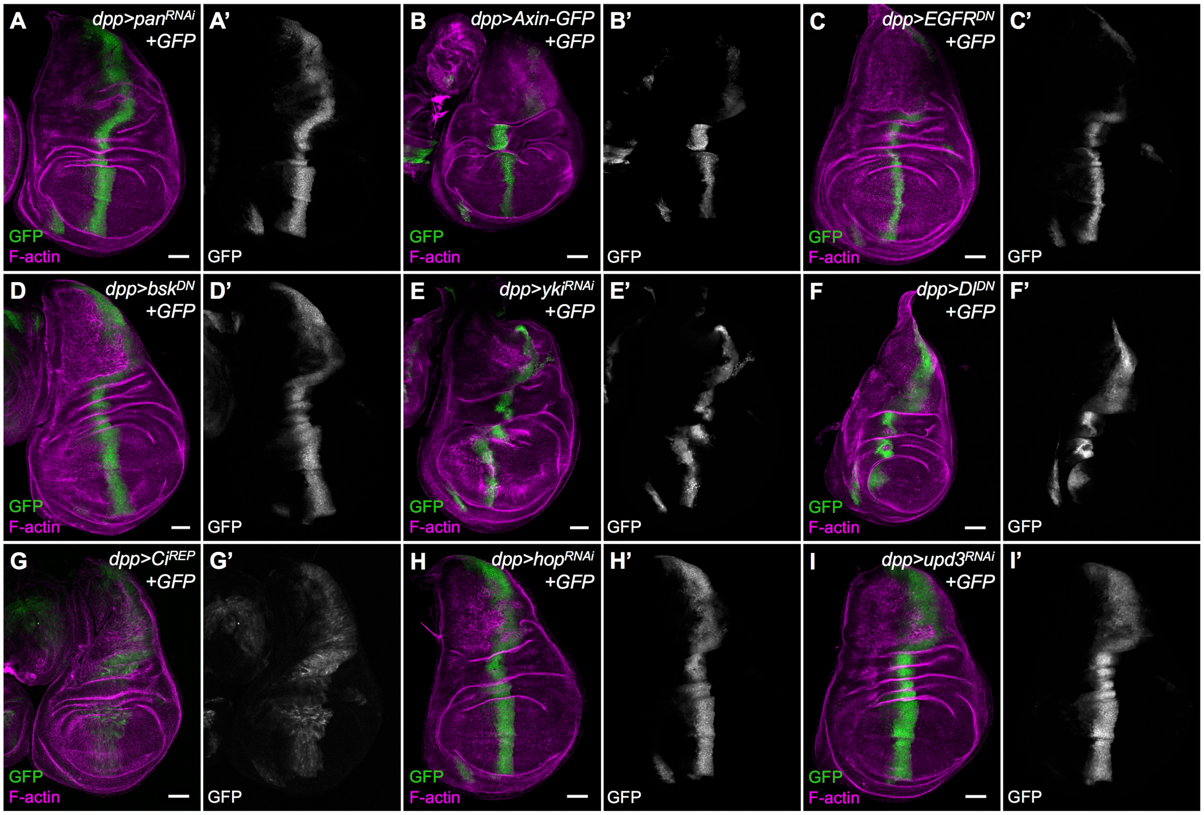

Figure S5: Phenotypes of signaling pathway disruptors used in study

The phenotypes of various UAS lines used in genetic analyses when driven by dpp-GAL4 were determined. Genetypes are indicated in panels. Discs were stained for GFP to reveal cells expressing transgenes and F-actin to reveal disc morphology. The following pathways were targeted with the indicated transgenes: Wg, using (A) *UAS-pan*^{RNAi} [dTCF] and (B) *UAS-Axin-GFP*; EGFR, using (C) *UAS-EGFR*^{DN}; JNK, using (D) *UAS-bsk*^{DN}; Hippo, using (E) *UAS-yki*^{RNAi}; Notch, using (F) *UAS-Dl*^{DN}; Hedgehog, using (G) *UAS-Ci*^{Rep}; JAK/STAT using (H) *UAS-hop*^{RNAi} and (I) *UAS-upd3*^{RNAi}.

Disease Models & Mechanisms • Supplementary information

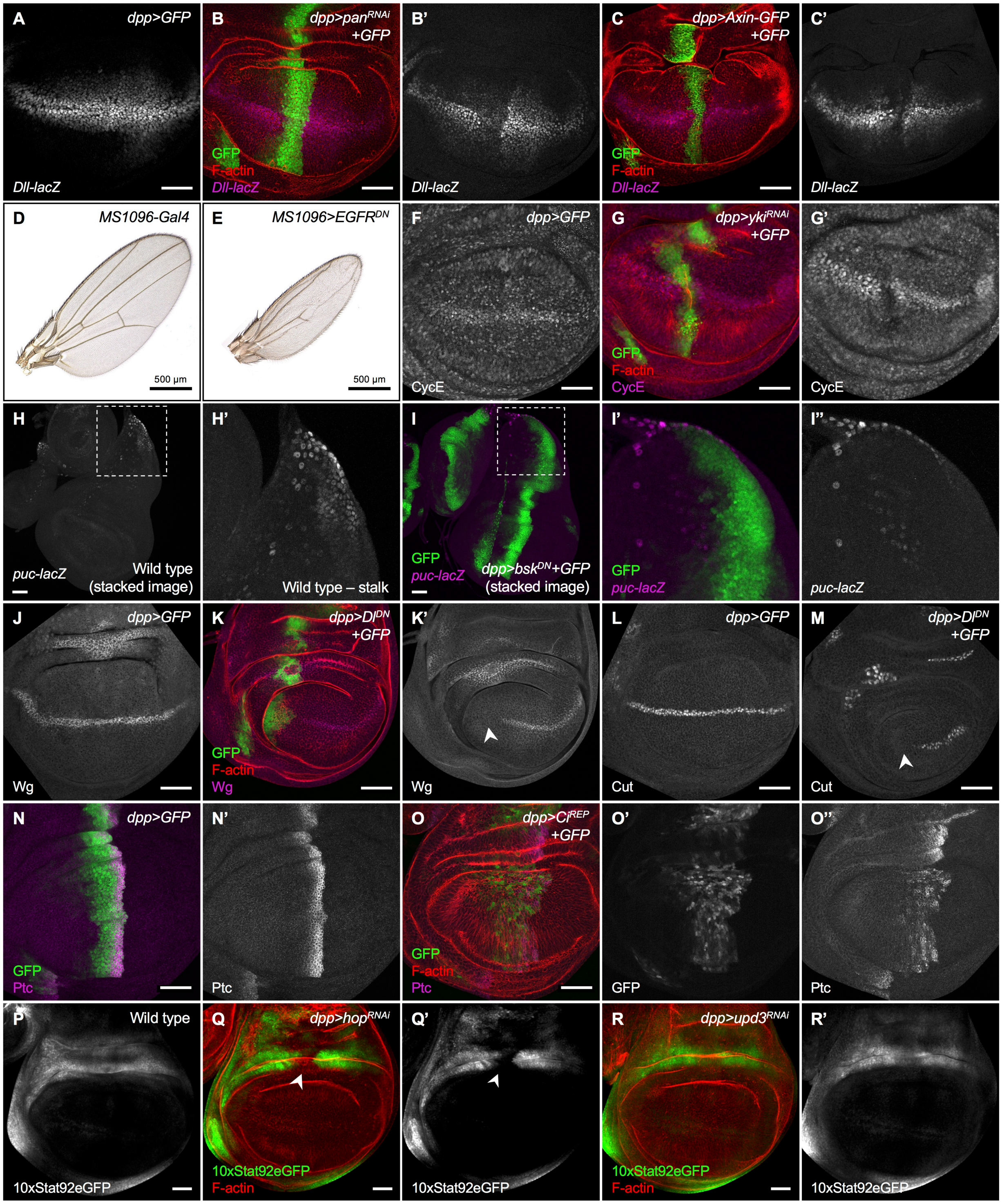
A dpp>GFP
Dll-lacZ
B dpp>pan^{RNAi} +GFP
GFP
F-actin
Dll-lacZ
B'
Dll-lacZ
C dpp>Axin-GFP +GFP
GFP
F-actin
Dll-lacZ
C'
Dll-lacZ
D MS1096-Gal4
500 µm
E MS1096>EGFR^{DN}
500 µm
F dpp>GFP
CycE
G dpp>yki^{RNAi} +GFP
GFP
F-actin
CycE
G'
CycE
H
puc-lacZ
Wild type (stacked image)
H'
Wild type – stalk
I
GFP
puc-lacZ
dpp>bsk^{DN}+GFP (stacked image)
I'
GFP
puc-lacZ
I''
puc-lacZ
J dpp>GFP
Wg
K dpp>Dl^{DN} +GFP
GFP
F-actin
Wg
K'
Wg
L dpp>GFP
Cut
M dpp>Dl^{DN} +GFP
Cut
N dpp>GFP
GFP
Ptc
N'
Ptc
O dpp>Ci^{REP} +GFP
GFP
F-actin
Ptc
O'
GFP
O''
Ptc
P Wild type
10xStat92eGFP
Q dpp>hop^{RNAi}
10xStat92eGFP
F-actin
Q'
10xStat92eGFP
R dpp>upd3^{RNAi}
10xStat92eGFP
F-actin
R'
10xStat92eGFP

Figure S6: Validation of signaling pathway disruption by reagents used in study.

To validate that all transgenes used in the study targeted their respective pathways, we examined either phenotypes or changes in target genes following GAL4-driven expression of either GFP or the indicated transgenes. Discs were stained for targets, GFP (to reveal cells expressing transgenes) and F-actin to reveal disc morphology:
(A) *dpp>GFP*, (B) *UAS-pan*^{RNAi} +GFP [dTCF] and (C) *UAS-Axin-GFP* +GFP stained for Wg target *Dll-lacZ*. (D) *MS1096-GAL4* control adult wing. (E) *MS1096>UAS-EGFR*^{DN} adult showing typical EGFR loss of function vein loss phenotype. (F) *dpp>GFP*, (G) *UAS-yki*^{RNAi} +GFP stained using antibody for Hippo target gene CycE. (H) Control wing disc stained for JNK target *puc-lacZ*. (H') Inset shows expression in stalk of disc. (I) *dpp>UAS-bsk*^{DN} +GFP stained to reveal reduced *puc-lacZ.* (J) *dpp>GFP* and (K) *dpp>UAS-Dl*^{DN} +GFP stained for the Notch target Wg. (L) *dpp>GFP* and (M) *dpp>UAS-Dl*^{DN} +GFP stained for the Notch target Cut. (N) *dpp>GFP* and (O) *UAS-Ci*^{Rep} +GFP stained to reveal Hedgehog target Ptc. (P) Control disc, (Q) *dpp>UAS-hop*^{RNAi} and (R) *dpp>UAS-upd3*^{RNAi} expressing the 10xStat92EGFP reporter for JAK/STAT signaling, stained for GFP.

Disease Models & Mechanisms • Supplementary information

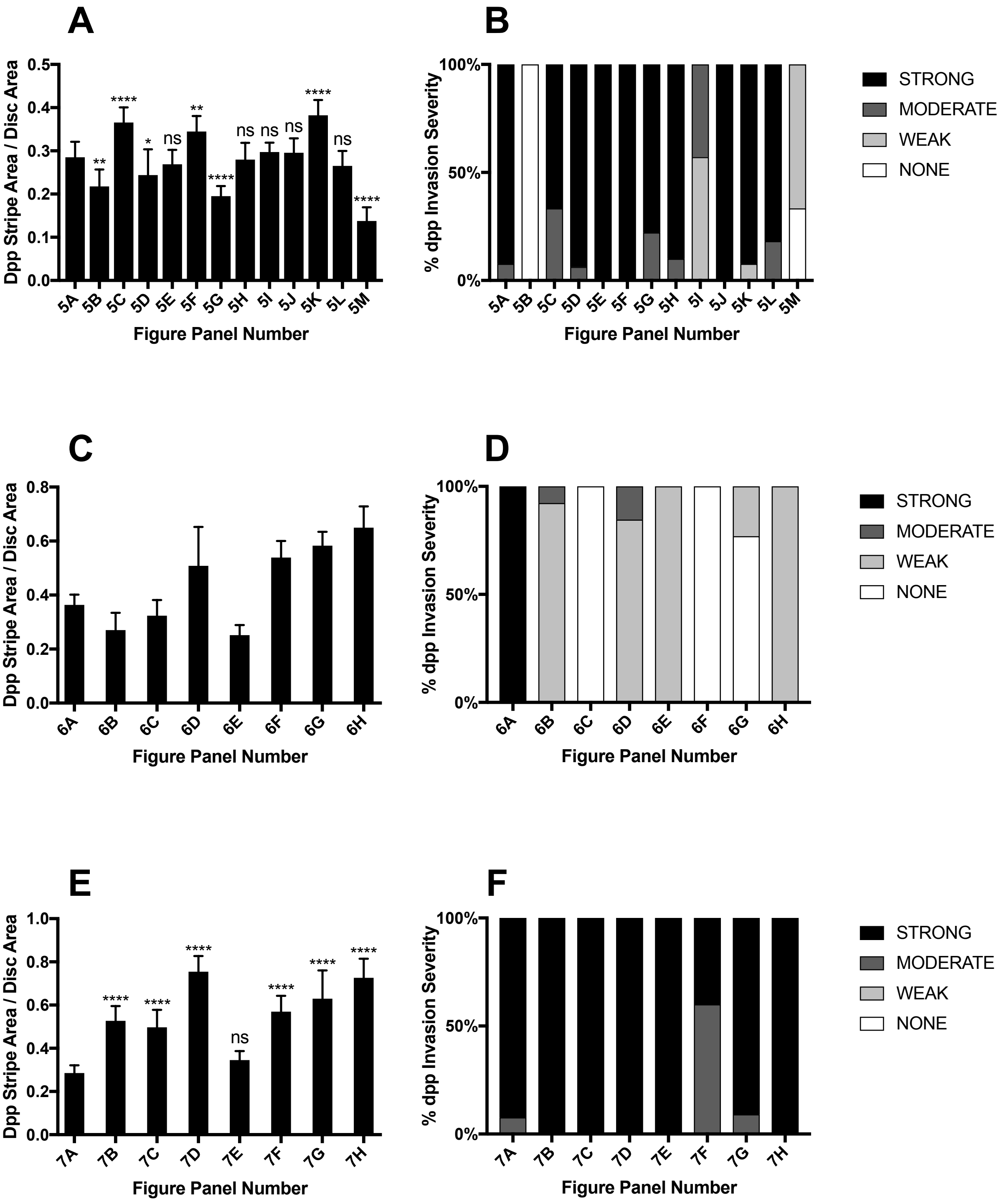

Disease Models & Mechanisms • Supplementary information

Figure S7: Quantification of proliferative areas in discs and degree of invasiveness in Figs. 5-7

Imaginal disc size measurements and invasiveness scoring were performed as described in methods. The genotypes in the following charts are:

Panels A-B

5A	*dpp>HA-hipk*^{3M}*, UAS-RFP, UAS-GFP*
5B	*dpp>HA-hipk*^{3M}*, UAS-hipk*^{RNAi}*, UAS-GFP*
5C	*dpp>HA-hipk*^{3M}*, UAS-pan*^{RNAi}*, UAS-GFP*
5D	*dpp>HA-hipk*^{3M}*, UAS-Axin-GFP, UAS-GFP*
5E	*dpp>HA-hipk*^{3M}*, UAS-EGFR*^{DN}*, UAS-GFP*
5F	*dpp>HA-hipk*^{3M}*, UAS-Bsk*^{DN}*, UAS-GFP*
5G	*dpp>HA-hipk*^{3M}*, UAS-yki*^{RNAi}*, UAS-GFP*
5H	*dpp>HA-hipk*^{3M}*, UAS-Dl*^{DN}*, UAS-GFP*
5I	*dpp>HA-hipk*^{3M}*, UAS-Ci*^{Rep}*, UAS-GFP*
5J	*dpp>HA-hipk*^{3M}*, UAS-hop*^{RNAi}*, UAS-GFP*
5K	*dpp>HA-hipk*^{3M}*, UAS-upd3*^{RNAi}*, UAS-GFP*
5L	*dpp>HA-hipk*^{3M}
5M	*dpp>HA-hipk*^{3M}*, UAS-yki*^{RNAi}*, UAS- Bsk*^{DN}

Panels C-D

6A	*dpp>HA-hipk*^{3M}*, UAS-GFP*
6B	*dpp>UAS-Arm*^{S10}*, UAS-GFP*
6C	*dpp>UAS-stat92E, UAS-GFP*
6D	*dpp>UAS-Ras*^{act}*, UAS-GFP*
6E	*dpp>UAS-eiger, UAS-GFP*
6F	*dpp>UAS-yki*^{S168A}*:GFP, UAS-GFP*
6G	*dpp>UAS-N*^{act}*, UAS-GFP*
6H	*dpp>UAS-Ci, UAS-GFP*

Panels E-F

7A	*dpp>HA-hipk*^{3M}*, UAS-RFP, UAS-GFP*
7B	*dpp>HA-hipk*^{3M}*, UAS-Arm*^{S10}*, UAS-GFP*
7C	*dpp>HA-hipk*^{3M}*, UAS-stat92E, UAS-GFP*
7D	*dpp>HA-hipk*^{3M}*, UAS-Ras*^{act}*, UAS-GFP*
7E	*dpp>HA-hipk*^{3M}*, UAS-eiger, UAS-GFP*
7F	*dpp>HA-hipk*^{3M}*, UAS-yki*^{S168A}*:GFP, UAS-GFP*
7G	*dpp>HA-hipk*^{3M}*, UAS-N*^{act}*, UAS-GFP*
7H	*dpp>HA-hipk*^{3M}*, UAS-Ci, UAS-GFP*

Disease Models & Mechanisms • Supplementary information

Supplementary Table 1

Signaling pathway reagents used in study

Pathway name	Loss of function	Gain of function
Wingless	*UAS-pan*^{RNAi} (TCF)<br>*UAS-Axin*	*UAS-Arm*^{S10}
EGFR/Ras	*UAS-EGFR*^{DN}	*UAS-Ras*^{act}
JNK	*UAS-bsk*^{DN}	*UAS-Eiger*
Hippo	*UAS-Yki*^{S168A}	*UAS-yki*^{RNAi}
Notch	*UAS-Delta*^{DN}	*UAS-Notch*^{act}
Hedgehog	*UAS-Ci*^{Rep}	*UAS-Ci*^{5M}
JAK/STAT	*UAS-hop*^{RNAi}<br>*UAS-upd3*^{RNAi}	*UAS-Stat92E*

See Methods for details on strains

Disease Models & Mechanisms • Supplementary information

Supplementary Table 2: Raw data

qPCR Ct, ΔCt, ΔΔCt , and 2^(-ΔΔCt) values for MDAMB231 cells transfected with either pCMV-myc (control) or pCMV-myc-dHipk (transfected)						
	Tested genes					
	HPRT control	HPRT transfected	dHipk control	dHipk transfected	ECAD control	ECAD transfected
Replicate #	Ct					
1	20.458896	20.45409838	N/A	24.61656284	25.48129463	28.54435476
2	22.92055257	22.6063563	N/A	23.47188314	24.72272491	28.18616803
3	23.66424179	23.38731893	N/A	24.24551392	19.63424428	24.56048902
	ΔCt					
1			Not Possible	0.865526835	5.022398631	8.090256373
2			Not Possible	1.199629784	1.802172343	5.579811732
3			Not Possible	0.858194987	-4.029997508	1.17317009
	ΔΔCt					
1						3.067857742
2						3.777639389
3						5.203167597
	2^(-ΔΔCt)					
1						0.119256703
2						0.744480933
3						0.02714504

Supplementary Table 3:

Migration RFU data normalized to the averag of the control		
	MDA-MB-231	
Replicate #	Control	pCMV-myc-dHipk
1	1.097433015	2.138842046
2	0.977562301	2.333286491
3	0.924957841	1.624554993
4	0.895780078	2.839919541
5	1.039014374	2.683526799
6	1.065205548	2.445585216
7	1.0151289	1.031069312
8	0.817120002	1.148883447
9	1.167736606	1.331502601
10	1.149188902	
11	1.096471069	
12	0.754339601	

Supplementary Table 4: Raw data

Proliferation measured by OD @ 540nm						
	HEK293		MCF7		MDAMB231	
Replicate #	Control	pCMV-myc-dHipk	Control	pCMV-myc-dHipk	Control	pCMV-myc-dHipk
1	1.1751	2.0414	0.8615	1.074	0.6322	1.0617
2	1.179	2.1293	0.8662	1.1075	0.6578	1.0742
3	1.1286	2.1211	0.8735	1.1129	0.647	1.0655

Supplementary Table 5: Raw data

dpp Stripe Area / Disc Area Ratio Data for Figure 5													
Replicate #	5A	5B	5C	5D	5E	5F	5G	5H	5I	5J	5K	5L	5M
1	0.282	0.289	0.294	0.321	0.250	0.336	0.176	0.248	0.270	0.239	0.418	0.243	0.100
2	0.357	0.209	0.355	0.250	0.238	0.352	0.191	0.325	0.324	0.263	0.335	0.332	0.113
3	0.241	0.214	0.377	0.315	0.316	0.305	0.221	0.292	0.266	0.301	0.358	0.262	0.141
4	0.277	0.169	0.391	0.314	0.233	0.349	0.162	0.229	0.300	0.281	0.430	0.236	0.111
5	0.303	0.218	0.334	0.282	0.291	0.384	0.176	0.317	0.307	0.310	0.369	0.307	0.135
6	0.235	0.206	0.413	0.158	0.235	0.386	0.182	0.213	0.301	0.333	0.394	0.226	0.127
7	0.306		0.339	0.235	0.297	0.336	0.220	0.307	0.313	0.297	0.341	0.301	0.155
8	0.276		0.407	0.241	0.258	0.334	0.195	0.303		0.350	0.329	0.238	0.184
9	0.297		0.392	0.222	0.318	0.389	0.225	0.265		0.296	0.400	0.275	0.167
10	0.330		0.386	0.303	0.255	0.278	0.220	0.298		0.322	0.427	0.257	0.087
11	0.294		0.347	0.162			0.191			0.262	0.419	0.239	0.156
12	0.236		0.353	0.223			0.223				0.384		0.179
13	0.274			0.166			0.195				0.366		
14				0.300			0.218						
15				0.255			0.190						
16				0.158			0.176						
17							0.147						
18							0.209						

Supplementary Table 6: Raw data

dpp Stripe Area / Disc Area Ratio Data for Figure 6								
Replicate #	6A	6B	6C	6D	6E	6F	6G	6H
1	0.388	0.229	0.445	0.711	0.213	0.569	0.531	0.622
2	0.389	0.302	0.395	0.688	0.249	0.557	0.635	0.605
3	0.308	0.250	0.287	0.544	0.208	0.464	0.546	0.713
4	0.366	0.225	0.295	0.789	0.226	0.449	0.546	0.681
5		0.231	0.295	0.427	0.287	0.511	0.530	0.653
6		0.234	0.315	0.466	0.313	0.558	0.642	0.584
7		0.186	0.271	0.441	0.246	0.581	0.602	0.734
8		0.334	0.262	0.438	0.234	0.608	0.668	0.641
9		0.369	0.395	0.381	0.291	0.657	0.503	0.521
10		0.230	0.375	0.592		0.500	0.614	0.593
11		0.230	0.303	0.373		0.490	0.554	0.801
12		0.301	0.291	0.345		0.527	0.615	
13		0.397	0.328	0.412			0.597	
14			0.361					
15			0.241					

Supplementary Table 7: Raw data

dpp Stripe Area / Disc Area Ratio Data for Figure 7								
Replicate #	7A	7B	7C	7D	7E	7F	7G	7H
1	0.282	0.506	0.552	0.769	0.324	0.577	0.722	0.671
2	0.357	0.543	0.474	0.854	0.403	0.652	0.771	0.838
3	0.241	0.649	0.670	0.761	0.379	0.610	0.850	0.818
4	0.277	0.556	0.410	0.790	0.354	0.647	0.576	0.544
5	0.303	0.519	0.456	0.749	0.346	0.562	0.614	0.630
6	0.235	0.583	0.506	0.806	0.325	0.531	0.561	0.748
7	0.306	0.545	0.419	0.621	0.365	0.472	0.370	0.796
8	0.276	0.546	0.568	0.689	0.337	0.506	0.613	0.681
9	0.297	0.526	0.432		0.265	0.474	0.726	0.729
10	0.330	0.335	0.484		0.303	0.666	0.549	0.774
11	0.294	0.482			0.402		0.569	0.763
12	0.236	0.494						
13	0.274	0.579						
14		0.555						
15		0.483						

Supplementary Table 8: Raw data

dpp Invasion Severity Data for Figure 5													
Severity	5A	5B	5C	5D	5E	5F	5G	5H	5I	5J	5K	5L	5M
Strong	12	0	8	15	10	10	14	9	0	11	12	9	0
Moderate	1	0	4	1	0	0	4	1	3	0	0	2	0
Weak	0	0	0	0	0	0	0	0	4	0	1	0	8
None	0	6	0	0	0	0	0	0	0	0	0	0	4

Supplementary Table 9: Raw data

dpp Invasion Severity Data for Figure 6								
Severity	6A	6B	6C	6D	6E	6F	6G	6H
Strong	4	0	0	0	0	0	0	0
Moderate	0	1	0	2	0	0	0	0
Weak	0	12	0	11	9	0	3	11
None	0	0	15	0	0	12	10	0

Supplementary Table 10: Raw data

dpp Invasion Severity Data for Figure 7								
Severity	7A	7B	7C	7D	7E	7F	7G	7H
Strong	12	15	10	8	11	4	10	11
Moderate	1	0	0	0	0	6	1	0
Weak	0	0	0	0	0	0	0	0
None	0	0	0	0	0	0	0	0

Disease Models & Mechanisms • Supplementary information
